# XGBoost-SHAP analysis prioritizes lower-frequency variants predicted to affect HNF4A binding motifs in pancreatic progenitors

**DOI:** 10.1371/journal.pgen.1012361

**Published:** 2026-11-04

**Authors:** Kelly A. Frazer, Benjamin Henson, Jennifer P. Nguyen, Wilfredo G. Gonzalez Rivera, Matteo D’Antonio

**Affiliations:** 1 Institute of Genomic Medicine, University of California San Diego, Gilman Dr, La Jolla, California, United States of America; 2 Department of Pediatrics, University of California, San Diego, La Jolla, California, United States of America; 3 Bioinformatics and Systems Biology Graduate Program, University of California, San Diego, La Jolla, California, United States of America; 4 Department of Biomedical Informatics, University of California, San Diego, La Jolla, California, United States of America; 5 Department of Medicine, Division of Metabolism, Endocrinology and Nutrition and UW Medicine Diabetes Institute, University of Washington, Seattle, Washington, United States of America; The Children’s Hospital of Philadelphia, UNITED STATES OF AMERICA

## Abstract

**Background:**

Noncoding genetic variants underlie many complex traits, yet predicting their regulatory impact in cell type-specific contexts remains a major challenge. Transcription factor binding is a primary mechanism through which noncoding variants influence gene regulation, but the relationships among chromatin accessibility, variant frequency, and transcription factor binding are complex and nonlinear, limiting the performance of traditional linear models.

**Methods:**

We generated single-nucleus ATAC sequencing data from seven induced pluripotent stem cell-derived pancreatic progenitor cell samples and identified 93,803 heterozygous single nucleotide polymorphisms overlapping accessible chromatin regions. Allele-specific effects (ASE) were quantified for each variant via binomial tests. We trained XGBoost, logistic regression, and regularized logistic regression models to predict allele-specific effects via chromatin read depth, peak score, variant minor allele frequency (MAF), and transcription factor binding prediction for five pancreatic transcription factors: HNF4A, HNF1A, NEUROD1, NKX6-1, and PDX1. Model performance was assessed by the area under the receiver operating characteristic curve (AUC) via fivefold cross-validation as well as using test and validation SNP sets. Shapley (SHAP) value analysis was used to interpret model predictions and to cluster variants on the basis of their regulatory feature contributions.

**Results:**

XGBoost significantly outperformed linear models, particularly at stringent allele-specific effect thresholds, consistent with its ability to capture nonlinear interactions among features. Chromatin read depth, peak score, and minor allele frequency were the strongest predictors. Stratification by read-depth and MAF deciles showed that XGBoost retained better overall predictive performance across deciles than the linear models, indicating that its advantage was not explained by these variables alone. Dimensionality reduction of variants on the basis of Shapley value profiles revealed seven clusters with distinct chromatin states and variant allele frequencies. These findings were supported by highly significant pairwise differences in allele frequency distributions across the seven clusters. One cluster was enriched for lower-MAF SNPs showing strong predicted interactions with HNF4A binding motifs. Because MAF and its SHAP contribution were inputs to the embedding, the lower-MAF identity of this cluster was expected; its high ASE enrichment and distinctive HNF4A interaction structure were the biologically informative findings. Combined Annotation Dependent Depletion (CADD) analyses further supported a stronger relationship between HNF4A-related predictions and predicted deleteriousness in this cluster.

**Conclusions:**

Integration of single-nucleus chromatin accessibility data with an interpretable XGBoost-SHAP framework provides a method for prioritizing noncoding variants and identifying nonlinear regulatory relationships in a specific developmental context. Within this framework, the stronger relationship between HNF4A-related predictions and CADD-predicted deleteriousness in the lower-MAF cluster is consistent with selective constraint on HNF4A-bound regulatory elements and suggests a hypothesis for how lower-frequency noncoding variation may contribute to intrafamilial phenotypic variability in HNF4A-related Maturity-Onset Diabetes of the Young (MODY1).

## Author summary

Most genetic variants linked to common diseases are located outside of coding genes, in regulatory regions that control the cell types and timing of gene expression. Identifying the noncoding variants that actually change gene regulation, and determining the underlying functional mechanism, remains difficult. Here we studied pancreatic progenitor cells, the developmental precursors of the insulin-producing cells of the pancreas, using single-nucleus ATAC-seq to measure chromatin accessibility in seven individuals. We built an interpretable machine-learning model (XGBoost) to predict which variants show allele-specific effects on chromatin accessibility, and used Shapley (SHAP) values to understand what the model learned. XGBoost outperformed linear models, consistent with its ability to capture nonlinear interactions among features. The model relied most heavily on chromatin accessibility measures, which reflect both biological accessibility and the read depth available to detect allele-specific effects. Grouping variants by their SHAP profiles revealed distinct predictive categories. We identified one particularly interesting group composed of variants with relatively lower allele frequencies whose predicted effects were associated with HNF4A sequence binding motifs. Although this group’s lower allele frequency was not an unexpected finding because allele frequency was one of the inputs used to cluster variants, its distinctive HNF4A interaction structure and high enrichment for allele-specific effects were biologically informative. CADD analyses further supported a stronger relationship between HNF4A-related predictions and predicted deleteriousness in this group. HNF4A is a transcription factor essential for pancreatic development and mutated in a form of monogenic diabetes (Maturity-Onset Diabetes of the Young, MODY1). These findings may provide insights into phenotypic variability among family members segregating MODY1. Our results show that even small single-cell datasets, combined with interpretable machine learning, can prioritize candidate regulatory variants for experimental follow-up.

## Introduction

Genome-wide association studies (GWASs) have identified thousands of loci linked to common diseases, but the majority of associated variants lie in noncoding regions and are believed to act through cell type-specific effects on gene regulation [1,2]. Hence, noncoding genetic variants play a central role in the genetic architecture of complex traits and diseases; however, predicting their regulatory impact remains a major challenge [3,4]. The functional interpretation of these variants could be elucidated through machine learning models that integrate both sequence-level and cell type-specific epigenomic regulatory information [5].

We, along with others, have demonstrated that altered transcription factor (TF) binding is a primary mechanism underlying the association between genetic variants and complex traits [6–9]. The transcription factors HNF1A, HNF4A, NEUROD1, NKX6–1, and PDX1 are relevant to the development and function of the pancreas and play crucial roles in pancreatic progenitor cells (PPCs). PDX1 and NKX6–1 are two primary transcription factors highly expressed in PPCs. Coexpression of PDX1 and NKX6–1 is a hallmark of the progenitors that give rise to functional beta cells, whereas NEUROD1 is an endocrine progenitor marker. Additionally, HNF1A and HNF4A are expressed in pancreatic progenitor cells and play vital roles in their specification, proliferation, and maintenance. Understanding how SNP alleles affect the active binding sites of these five TFs could reveal important variations in regulatory activity during early pancreas development [10,11].

Recent advances in single-cell epigenomic profiling, particularly single-nucleus assays for transposase-accessible chromatin using sequencing (snATAC-seq) [12], have enabled the identification of cell type-specific active chromatin states. In ATAC-seq data, the read depth and peak score represent quantitative measures of chromatin accessibility. Regions with greater accessibility, and therefore greater read depth, correspond to active regulatory elements where TFs are bound, whereas regions with lower accessibility represent inactive or repressed chromatin states. Thus, the association of different SNP alleles with snATAC-seq read depth and peak score is central to understanding how TF-driven regulatory activity varies across individuals. Of note, read depth also determines statistical power to detect allele-specific effects, so its biological accessibility and detection-power roles must be considered together. snATAC-seq data provide valuable insights into the regulatory landscape of specific cell types, developmental stages and diseases and offer a unique opportunity to assess allele-specific effects (ASEs) of genetic variation on chromatin accessibility and presumed downstream gene regulation. However, snATAC-seq studies typically include small sample sizes (<10 individuals), and the systematic use of snATAC-seq to construct predictive models of variant function remains underexplored.

Emerging machine learning (ML) and artificial intelligence (AI) models, including frameworks trained on genomic and epigenomic data, are starting to advance our ability to prioritize candidate causal variants by modeling complex regulatory mechanisms and predicting variant effects on chromatin accessibility, TF binding, and gene expression [13–15]. These tools allow fine-mapping of GWAS loci and the assignment of functions to noncoding single nucleotide polymorphisms (SNPs) with increasing precision. New ML/AI models, such as Enformer and DNABERT, can capture broad regulatory contexts and can be used to generally predict whether a genomic region is regulatory, but are still inaccurate at determining the effects of single base pair changes [16,17]. Furthermore, currently available tools mostly offer a black box approach and do not allow for an interpretation of the variables used in the prediction. ML methods such as eXtreme Gradient Boosting (XGBoost) [18], combined with Shapley (SHAP) analysis [19], have been increasingly used in genomics and provide an interpretable account of how each variable contributes to predictions, including nonlinear feature interactions [17,20–22]. Importantly, these SHAP contributions represent predictive associations rather than causal effects and therefore require orthogonal computational support and/or experimental validation before causal interpretation. While interpretable ML approaches for genomics are an active area of research [23], the integration of single-cell ATAC-seq accessibility features with allele-specific effects and TF binding predictions for the purpose of regulatory variant prioritization in a defined cell type has not been previously described. Our approach is specifically designed to work with small-sample snATAC-seq cohorts (n = 7) and leverages cell type-specific accessibility as the primary biological signal, distinguishing it from approaches based on large-scale population data or sequence models alone. Previous studies have demonstrated the value of combining deep learning with SHAP-based attribution to extract genetic insights from genomic models [20], established frameworks for gene expression inference via deep learning, highlighted the interpretability challenge for sequence-to-function models [17,22], and provided a comprehensive treatment of deep learning approaches for genomics [23]. The present study advances these prior methods by applying allele-level SHAP attribution directly to single-nucleus chromatin accessibility data from pancreatic progenitor cells, enabling variant prioritization in a biologically specific developmental context without the need for large-scale sequence training corpora.

Here, we used snATAC-seq data generated from 7 induced pluripotent stem cell (iPSC)-derived pancreatic progenitor cell (iPSC-PPC) samples to develop and evaluate an XGBoost computational framework to predict the regulatory function of noncoding variants (Fig 1). Our study revealed that XGBoost can capture nonlinear relationships among read depth, peak score, SNP minor allele frequencies (MAFs), and TF binding predictions, while SHAP analysis provides an interpretable view of the feature contributions underlying its ASE predictions. We found that UMAP clustering of SNPs on the basis of their Shapley (SHAP) values for each feature was influenced mainly by read depth, SNP MAF, and peak score. SNPs in putative functional elements with high read depth and peak scores were more likely to show ASE. One SHAP-defined group was distinguished by strong predicted interactions involving HNF4A binding-motif scores, high ASE enrichment, and a stronger relationship between HNF4A-related predictions and CADD-predicted deleteriousness, consistent with selective constraint on HNF4A-bound regulatory elements.

**Fig 1:**
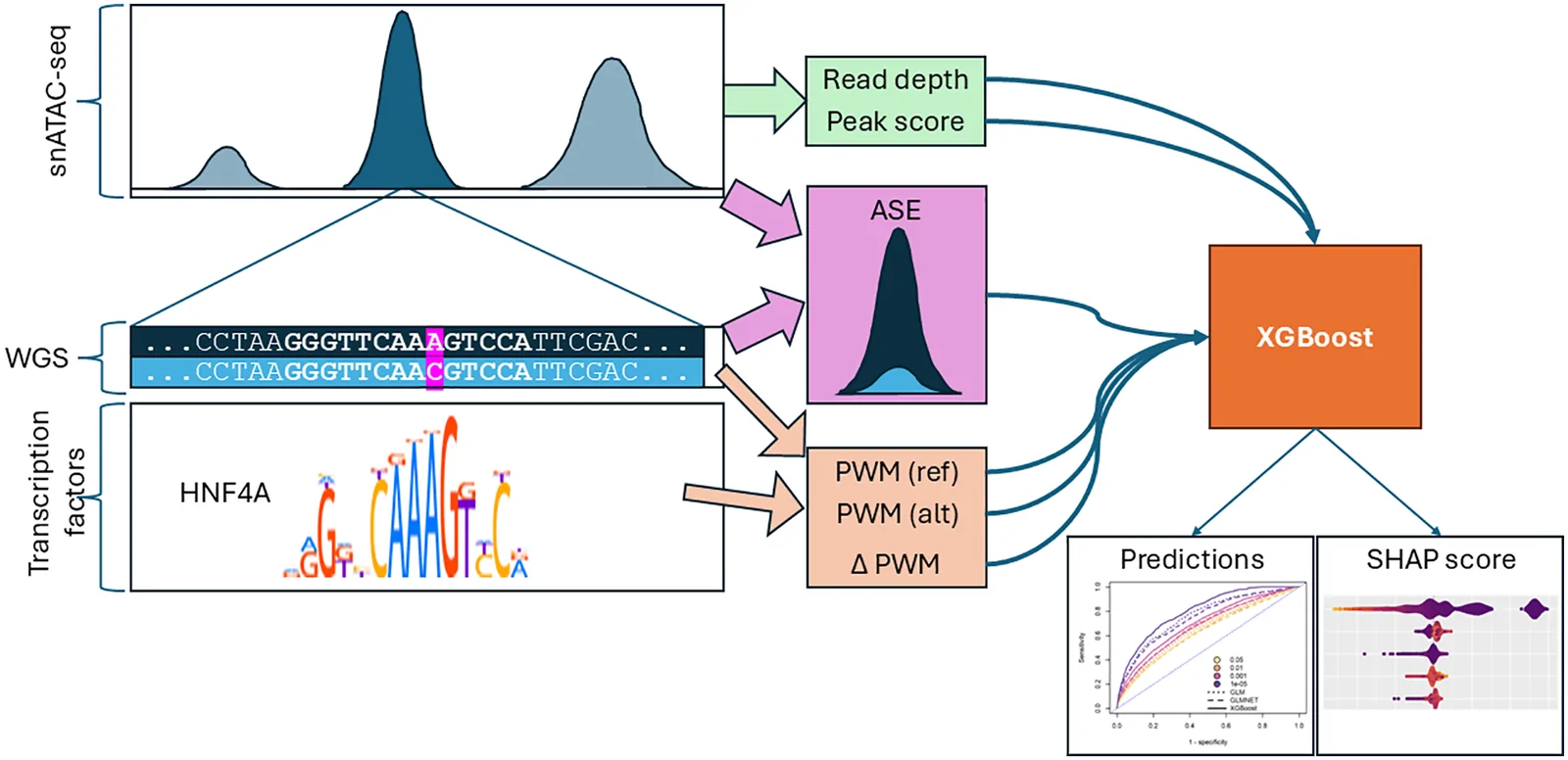
Model overview. Architectural schematic depicting the workflow. On the left, the primary data are shown, including snATAC-seq, whole-genome sequence information (SNP alleles: A/C) and a PWM associated with the HNF4A TF. The whole-genome sequence information was used to identify SNPs across the genome in each of the seven individuals included in the study. In the middle, the model features and training outcome are shown; for each peak in the snATAC-seq data, the read depth and peak score were obtained. Variant MAF was also included as a model feature. The snATAC-seq data were used to identify which SNPs presented allele-specific effects (ASE) on chromatin accessibility, depicted as the dark blue allele having greater sequence coverage than the light blue allele. Observed ASE status served as the model-training outcome. For five TFs, the PWM (ref), PWM (alt) and delta PWM were calculated at each SNP. On the right, the XGBoost model with predictions and SHAP analysis is shown. After training, XGBoost performance in distinguishing SNPs with and without ASE was evaluated using repeated fivefold cross-validation as well as test and validation SNP sets.

## Methods

### Ethics statement

The recruitment of iPSCORE individuals was conducted in accordance with the principles of the Declaration of Helsinki and approved by the Institutional Review Boards of the University of California, San Diego, and the Salk Institute (Project no. 110776ZF). Written informed consent was obtained from all participants. The genetic data used in this manuscript have been published previously and deposited in dbGaP (phs000924 and phs001325).

### iPSC-PPC derivation

We derived and cryopreserved pancreatic progenitor cells (PPCs) from 7 iPSC lines (iPSC-PPCs) via the STEMdiff Pancreatic Progenitor Kit (StemCell Technologies), as described in detail in S1 Text. We have previously shown that these iPSC-PPCs serve as model systems for early pancreas development and express key transcription factors, including HNF1A, HNF4A, NEUROD1, NKX6–1, and PDX1, which are pivotal for pancreas lineage specification and differentiation [6].

### Generation of snATAC-seq

*Library generation*: Immediately after thawing, cells from four of these cryopreserved iPSC-PPC samples were pooled (ATAC_Pool_1), and cells from the other 3 iPSC-PPC samples were pooled (ATAC_Pool_2). Nuclei from the two pools were isolated according to the manufacturer’s recommendations (Manual CG000169, Rev B), transposed, and captured independently according to the manufacturer’s recommendations (Manual CG000168, Rev B). All single nuclei were captured via a 10x Chromium controller (10x Genomics) according to the manufacturer’s specifications and manual (Manual CG000168, Rev B). The cells for each pool were loaded on an individual lane of a Chromium Chip E. Libraries were generated via the Chromium Single Cell ATAC Library Gel & Bead Kit (10x Genomics) following the manufacturer’s manual (Manual CG000168, Rev B). The sample index PCR material was amplified for 11 cycles.

*Sequencing*: Libraries were sequenced via a custom program (50-8-16-50 Paired-End) on a HiSeq 4000 platform. Specifically, two libraries comprising the seven iPSC-PPC samples (ATAC_Pool_1, including PPCs from four subjects, and ATAC_Pool_2, including PPCs from three subjects) were each sequenced in an individual lane.

### Processing snATAC-seq data

*Raw data processing.* For the two snATAC-seq pools (ATAC_Pool_1 and ATAC_Pool_2), we retrieved FASTQ files and used CellRanger V2.0.0 (https://support.10xgenomics.com/) to align files to the hg19 genome via *cellranger-atac count* with default parameters. NarrowPeaks were called via the MACS2 command *macs2 callpeak --keep-dup all --nomodel --call-summits* [24] on the BAM files merged from the two snATAC-seq pools, and 288,813 peaks were detected. Peaks called on noncanonical chromosomes or the mitochondrial genome were removed, leaving 280,079 peaks. Using these peaks, each snATAC-seq pool was reanalyzed via *cellranger-atac* to generate single-nucleus peak counts. To integrate the two datasets for downstream analyses, we performed Signac integration [25] by first applying normalization (*RunTFIDF*) and linear dimensional reduction (*FindTopFeatures* and *RunSVD*) on each dataset. We then identified a random subset of 20,000 peaks and computed a set of integration anchors between the two pools (*FindIntegrationAnchors* for 2,000 anchors). The two snATAC-seq datasets were integrated via *IntegrateData,* and the 2–30 most significant dimensions were calculated via dimension reduction analyses. Finally, on the integrated dataset, dimension reduction was applied (*RunSVD* for 30 singular values), and single cells were visualized via UMAP (*RunUMAP* on 2:30 dimensions). Clusters were identified via the SNN-graph method via *FindNeighbors* and *FindClusters*. To remove low-quality cells, we removed cells that satisfied any of the following criteria: 1) number of peak region fragments < 2,000 or > 20,000, 2) percentage of reads in peaks < 40%, 3) nucleosome signal > 1.5, or 4) TSS enrichment score < 2.5. Furthermore, we removed cells that did not visually belong to a cluster (i.e., cells that are scattered between two distinct clusters). We performed iterative clustering until we did not observe significant outliers of single cells. After filtering, 26,026 nuclei remained, and clustering resolutions of 0.1, 0.15, and 0.2 were tested. Detailed per-sample snATAC-seq quality control statistics, including sequencing depth, number of fragments, and mean reads per cell, are provided in S1 Table and are consistent with high-quality single-nucleus ATAC-seq profiles.

*Demultiplexing.* Because we sequenced pooled samples (ATAC_Pool_1, including PPCs from four subjects, and ATAC_Pool_2, including PPCs from three subjects), we applied Demuxlet [26] to the two snATAC-seq pools to assign each nucleus to its associated subject, using the variants from the 1000 Genomes Phase 3 panel that are biallelic and have minor allele frequencies between 45% and 55%.

### Annotation of iPSC-PPC nuclei in snATAC-seq using chromVAR

To determine the cell types within the two pooled snATAC-seq datasets, we used chromVAR [13] in the Signac pipeline to identify transcription factor motifs from the JASPAR 2022 database [27] that were enriched for accessible chromatin in each cluster. Specifically, we used the *RunChromVAR* function in Signac and the hg19 reference (BSgenome.Hsapiens.UCSC.hg19 from https://bioconductor.org/packages/BSgenome.Hsapiens.UCSC.hg19/) to compute a deviation z score for each motif in each cell. To annotate the cell types, we examined the motif activities of transcription factors with known developmental or pancreatic functions (S1 Fig): TFAP2A/B (mesendoderm), GATA4/6 (PDX1 + progenitors), HNF4A, FOXA1/2, PDX1, NKX6–1 (NKX6–1 + progenitors), PAX4/6, RFX1/3, HNF1A, MAFA, NKX2–2, and NEUROD1 (endocrine), and ETV1, ETS1, and ETS2 (early ductal). To validate our annotations, we compared the motif activities to their gene expression in scRNA-seq [6] via the same z-normalization method. A resolution of 0.1 was used for downstream analyses. Our annotations revealed that the iPSC-PPC samples were largely composed of NKX6–1 + progenitors (S1 Fig). While a small proportion of cells corresponded to non-NKX6–1 + populations (mesendoderm, early endocrine, endocrine, and early ductal; comprising approximately 7.5% of all 26,026 cells), these populations were not excluded from the ASE analyses. The inclusion of all nuclei is appropriate for our study because (1) the predominant population (92.5%) corresponds to NKX6–1 + progenitors, ensuring that ASE signals are predominantly reflective of this cell type; (2) our ASE framework relies on per-SNP binomial tests at individual variant positions, which are not sensitive to the presence of a minor fraction of off-target cell types; and (3) the chromatin accessibility features (read depth and peak score) at each SNP position are derived from merged data, and inclusion of the small minority population contributes minimally to overall accessibility measurements.

### Allele-specific effects on snATAC-seq peaks

We filtered the 280,079 snATAC-seq peaks to include peaks on autosomal chromosomes, with MACS2 scores ≥ 100, and outside of ENCODE blacklist regions (hg19-blacklist.v2.bed.gz: https://github.com/Boyle-Lab/Blacklist/blob/master/lists/hg19-blacklist.v2.bed.gz). We intersected the resulting 203,895 peaks with the SNPs that were heterozygous in at least one of the seven iPSC-PPC samples that underwent snATAC-seq. Variant information was obtained from our previously published whole-genome sequencing (dbGaP: phs001325: https://www.ncbi.nlm.nih.gov/projects/gap/cgi-bin/study.cgi?study_id=phs001325) [28–30]. We obtained 109,405 SNPs with read depths ≥ 20 and minor allele frequencies (MAFs) ≥ 5% in the iPSCORE cohort [28] that were heterozygous in at least one sample, which we divided into: 1) 60,780 SNPs heterozygous only in one sample (singletons), and 2) 48,625 SNPs heterozygous in multiple samples (multiplets). We calculated allele-specific effects (ASEs) for each SNP independently in each heterozygous sample via a two-sided binomial test with the null hypothesis that both alleles were equally likely to be observed (p = 0.5). We removed 15,602 multiplets with inconsistent effects, which had the sign of ${log}_{\text{2}}\left(\frac{{reads}_{REF}}{{read}_{ALT}}\right)$ inconsistent across heterozygous samples, and 33,023 were retained for downstream analyses. This resulted in 93,803 heterozygous SNPs that were heterozygous only in one sample or heterozygous in multiple samples and that had consistent allelic effects across all heterozygous samples. FDR correction was performed on a per-sample basis via Benjamini‒Hochberg’s method on the 93,803 heterozygous SNPs (17,199–36,517 tests per sample).

We did not apply WASP [31] or other allele-aware filtering methods because high-quality ATAC-seq peaks with sufficient read depth allow accurate estimation of allele-specific effects. Prior work has shown that reference bias is minimal in regions with strong chromatin accessibility [32]. In addition, our analyses rely on direct comparisons of REF and ALT position weight matrix (PWM) scores (see below), which inherently account for potential reference allele bias.

### Effects of SNPs on transcription factor binding sequences

For each of the 93,803 heterozygous SNPs, we obtained the surrounding 20 bp genomic sequence (SNP at position 0; positions −10 to +9) via bedtools-2.27.1 *getfasta* and generated two sequences (reference and alternate alleles) in FASTA format with R v4.2.1. We next obtained position weight matrices (PWMs) in JASPAR format [33] for the following five pancreatic progenitor-relevant transcription factors: HNF1A, HNF4A, NEUROD1, NKX6–1, and PDX1. We ran FIMO (Find Individual Motif Occurrences), which is part of the MEME suite 4.12.0 [34], on both reference and alternate sequences to obtain the binding affinity within each 20 bp region for each of the five individual transcription factors. FIMO was run using the default p value threshold of 1e-4 to report significant motif matches, which is consistent with standard practice in TF binding motif analysis. For each transcription factor and each 20 bp region, we retained only the highest PWM score for both the reference and alternate allele sequences.

PWM scores provide a well-established, mechanistically interpretable estimate of TF binding affinity at single-nucleotide resolution, making them ideally suited for quantifying how a base substitution alters TF–DNA interactions. Because TF binding motifs are short (typically 6–20 bp), we extracted a 20 bp window centered on the SNP to ensure that the analyzed sequence fully captures the TF binding site potentially affected by the variant. Importantly, expanding the sequence window beyond 20 bp would incorporate flanking regions that do not contribute to PWM-based TF binding estimates and are not altered by the SNP itself. This would dilute the allele-specific signal and introduce noise unrelated to TF–DNA affinity changes. Thus, the 20 bp window was chosen deliberately to maintain mechanistic interpretability and to ensure that the PWM scores directly reflect the SNP’s predicted impact on TF binding.

### Running predictions

Predictions were run via the function *train* from the caret R package 7.0.1 with fivefold cross-validation using the following covariates: snATAC-seq read depth, peak score, SNP MAF in the iPSCORE population [28] and the transcription factor 20 bp region-specific information. We ran:

- Three types of models: logistic regression (GLM), elastic net regularized logistic models (GLMNET) and XGBoost. We optimized both GLMNET and XGBoost: for GLMNET, we attempted different alpha and lambda parameters to test both LASSO and ridge regressions; for XGBoost, we optimized the number of rounds, maximum tree depth, gamma, column and row sampling. The optimizations were performed via the *expand.grid* function in caret. Specifically, the XGBoost hyperparameter search space includes nrounds ∈ {50, 100, 150}, max_depth ∈ {3, 6, 9}, eta (learning rate) ∈ {0.01, 0.1, 0.3}, gamma ∈ {0, 0.1, 0.2}, colsample_bytree ∈ {0.5, 0.75, 1.0}, and subsample ∈ {0.5, 0.75, 1.0}. The combination yielding the highest mean ROC AUC across fivefold cross-validation resamples on the training set was selected as the final model configuration. Feature selection was based on nonzero variance across training examples; features with near-zero variance were excluded prior to model fitting. SHAP analyses on XGBoost models were performed via Shapviz 0.9.7 and SHAPforxgboost 0.1.3 in R.
- Four ASE thresholds: Outcome was defined as positive for all SNPs whose ASE p value was more significant than a certain threshold. We used the following thresholds for p values: 0.05, 0.01, 0.001 and 1e-5.
- We tested each of the five transcription factors independently and built a model combining all five transcription factors. For each transcription factor, we used three variables: the PWM score for the reference allele, the PWM score for the alternate allele, and the difference between the PWM scores for the reference and alternate alleles (PWM delta).
- The peak score, calculated via MACS2, is a metric computed during peak calling that reflects the signal intensity, enrichment over background, and statistical confidence of that peak.
- Read depth at each variant position provides information regarding the power of the ASE tests at the position.
- SNP MAF provides information about the potential impact on ASE, as it is well known that low-frequency variants tend to have stronger phenotypic effects [35]. Notably, all variants analyzed had a MAF ≥ 5% in the iPSCORE cohort [28]; variants below this threshold were excluded from the analysis. The MAF was used as a continuous predictor variable in all the models, and no genotype imputation was performed.

We also tested the following models:

1. Using only chromatin accessibility features (snATAC-seq read depth and peak score) and the MAF of each SNP
2. Using only transcription factor data (PWM scores).
3. An additional 20 TFs relevant to pancreatic development, pancreatic function and diabetes were used to investigate whether expanding the number of TFs could enhance the predictive performance of the model (S2 Table, S2 Fig). By comparing the area under the receiver operating characteristic curve (ROC AUC) when only the five TFs were used with the expanded set of 25 TFs, we concluded that including the additional 20 TFs did not improve the predictive accuracy of the models.

One potential issue with prediction analysis is class imbalance, as only a small proportion of variants have ASE. We address this potential source of bias in multiple ways:

1. We selected evaluation metrics that are informative under class imbalance, specifically the ROC AUC and AUPRC (area under the precision‒recall curve). The AUPRC is particularly appropriate for imbalanced datasets. Both metrics are reported across all the models and thresholds in S3 Table.
2. We tested three fundamentally different model classes (GLM logistic regression, GLMNET regularized regression, and XGBoost gradient boosting), providing a systematic comparison of model types rather than relying on a single approach.
3. We did not apply explicit resampling; instead, we relied on evaluation metrics appropriate for imbalanced data (ROC AUC and, in particular, AUPRC) and on comparisons across model classes. We note that XGBoost’s internal regularization mitigates overfitting rather than class imbalance per se.

### Model evaluation and comparison

Predictive performance was assessed via the receiver operating characteristic (ROC) area under the curve (AUC), which was computed via fivefold cross-validation via the caret R package 7.0.1. For each model, resampling-based estimates of the ROC AUC were obtained.

To compare different model types (XGBoost, GLM, GLMNET) at a given ASE threshold, we evaluated each model on three sets of SNPs, 1) the “training set” (80% of the 60,780 individual heterozygous SNPs; N = 48,624); 2) the “test set” (remaining 20% of the 60,780 heterozygous SNPs; N = 12,156); and 3) the “validation set” (the 33,023 SNPs heterozygous in multiple individuals). The difference in AUC between two models (for example, XGBoost versus a linear model) was tested with a paired bootstrap test and the DeLong test as implemented in the pROC R package, both of which account for the same SNPs being scored by both models and therefore avoid the shared-training-data dependence of the fold-wise t-test; 95% confidence intervals for the AUC difference were obtained from 10,000 stratified bootstrap resamples of the test set of SNPs. We did not base model comparisons on paired t tests of cross-validation resamples, because cross-validation folds share training data and are therefore not independent, which can render such tests anti-conservative.

### ChromHMM analysis

We obtained BED files with chromHMM coordinates for seven pancreatic tissues (six adult and one iPSC-derived) from EpiMap (https://compbio.mit.edu/epiFmap/) [36,37]. We used *bedtools intersect* [38] to obtain the overlap of each SNP with chromatin states in each tissue.

## Results

### XGBoost models predict ASE outcomes better than linear models do

As a test biological system, we first performed snATAC-seq on iPSC-PPC samples derived from seven individuals. Analysis of the snATAC-seq samples revealed that >90% of the cells corresponded to a single population of PPCs that expressed the PDX1 and NKX6–1 TFs at high levels (S1 Fig). We used these samples, combined with the genotype information of the seven individuals [28], in two ways: 1) we characterized the ASE of each heterozygous SNP overlapping the snATAC-seq peaks; we used the ASE values as the “outcome” phenotype in the models described below; and 2) we used FIMO [34] to determine the effects of each variant on position weight matrices (PWMs) and whether the variant is likely to impact the transcription factor binding activity of five pancreatic progenitor-relevant transcription factors (HNF1A, HNF4A, NEUROD1, NKX6–1, and PDX1). We identified 93,803 SNPs heterozygous in at least one of the seven individuals located in snATAC-seq peaks, which we tested for ASE via a binomial test, and defined ASE across four p value thresholds (ranging between 0.05 and 1e-5). The number of SNPs reaching significance varied substantially across thresholds: approximately 17,064 SNPs presented an ASE at p < 0.05 (per-sample basis prior to FDR correction), 9,378 presented an ASE at p < 0.01, 5,123 presented an ASE at p < 0.001, and 2,188 presented an ASE at p < 1e-5. This distribution reflects the expected range of effect sizes across heterozygous variants in accessible chromatin regions. We divided the 93,803 heterozygous SNPs into three sets, which we used to develop machine learning models to predict variants’ effects on the ASE phenotype: 1) we used 80% of the 60,780 SNPs that were heterozygous in only one individual as the “training set” (N = 48,624); 2) the remaining 20% of these SNPs were used as the “test set” (N = 12,156); and 3) the 33,023 SNPs heterozygous in multiple individuals were used as the “validation set”.

First, we selected PDX1, a pancreatic development-specific TF that is expressed at high levels in all pancreatic progenitors [39], and investigated three model types to determine which best predicts ASE: 1) generalized linear model (GLM logistic regression); 2) regularized generalized linear model using penalized maximum likelihood (GLMNET), including L1 penalties (LASSO), L2 penalties (ridge) and elastic net regression (combining L1 and L2 penalties); and 3) a nonlinear effect model (XGBoost). As an outcome, we used ASE at different p value thresholds (between 0.05 and 1e-5) and considered all heterozygous SNPs passing the p value threshold as “positives” and the others as “negatives”. Notably, the majority of positive heterozygous SNPs did not overlap with a PDX1 TF binding site. The resulting datasets are inherently imbalanced, with ASE-positive SNPs constituting a minority of the total (ranging from approximately 10–20% positive at p < 0.05 to fewer than 3% at p < 1e-5). We address this class imbalance by evaluating model performance via the area under the ROC curve (ROC AUC) and the area under the precision‒recall curve (AUPRC), both of which are informative under imbalanced class conditions. We did not apply oversampling or undersampling procedures. We used read depth, peak score, variant minor allele frequency (MAF) in the iPSCORE population [28], the PWM value associated with the PDX1 reference and alternate alleles, and the PWM delta between the PDX1 reference and alternate alleles as covariates. We found that at loose ASE p value thresholds (p = 0.05), the three different PDX1 models were comparable, and all had low accuracy, with areas under the receiver operating characteristic curve (ROC AUC) between 0.597 (GLMNET) and 0.606 (XGBoost, cross-validation on the training set; Fig 2A, S3 Table). At more stringent ASE p value thresholds (p = 1e-5), we observed significantly better predictions for all the PDX1 models and found that XGBoost had better accuracy than the other models did (XGBoost vs GLM: ΔAUC = 0.0346, 95% bootstrap CI 0.0154-0.0545, bootstrap p = 5.45e-4, DeLong p = 4.83e-4; and XGBoost vs GLMNET: ΔAUC = 0.0569, 95% CI 0.0370-0.0777, bootstrap p = 2.44e-8, DeLong p = 2.55e-8). Results using the test and validation SNP sets similarly showed that the PDX1 XGBoost model performed better than either of the PDX1 linear models, and the best SNP ASE classifications were obtained using the most stringent ASE p-value threshold (p = 1e-5) (S3A, S3B Fig).

**Fig 2:**
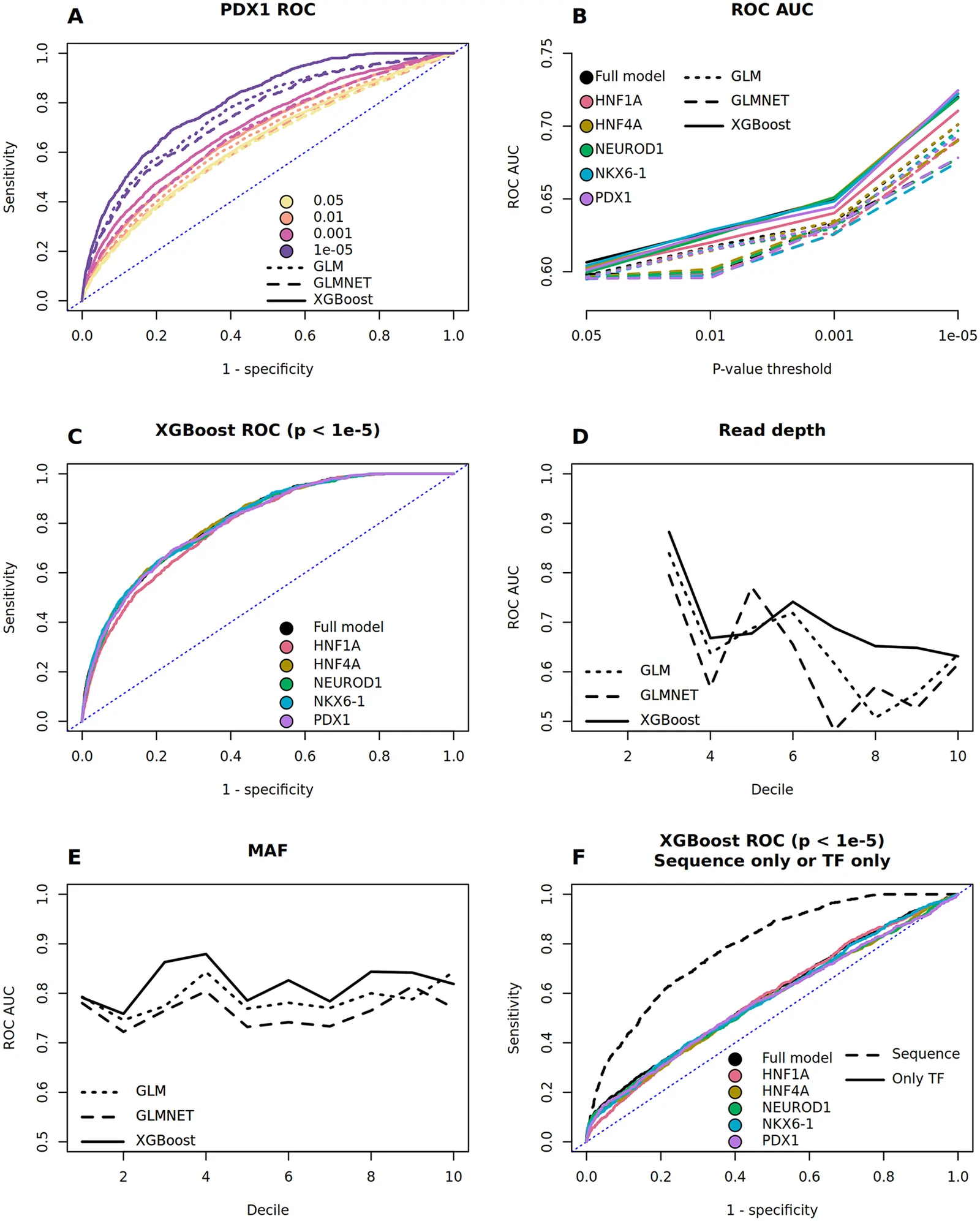
XGBoost improves predictive performance compared with linear models. A, C) ROC curves for A) predictions using only PDX1 at multiple ASE p value thresholds and models and C) for the “Full” XGBoost model including chromatin-accessibility features (snATAC-seq read depth and peak score) and SNP MAF and all five TFs as well as for each TF independently at an ASE p value threshold = 1e-5. The ROC curves calculated on the cross-validated training set are shown here. The test and validation SNP sets are shown in S3 Fig. B) ROC AUCs at different p value thresholds for all the models and all the transcription factors. D,E) Line plots showing the ROC AUC stratified by read depth (D) and MAF (E). In (D), the first two deciles do not include any variants with ASE, so no ROC AUC was calculated. The plots show that XGBoost had better overall predictive performance across the read-depth and MAF deciles than the linear models. F) ROC curves for models built using only transcription factor information (solid lines) or using only chromatin-accessibility features (snATAC-seq read depth and peak score) and SNP MAF (dashed line). In the panel, “Sequence” refers to these accessibility and frequency features rather than the underlying DNA sequence. “Full model” indicates the model built using the PWMs for all five TFs together with the chromatin-accessibility and MAF features.

We next investigated the three model types with all five TFs (HNF1A, HNF4A, NEUROD1, NKX6–1, and PDX1) independently as covariates, as well as a combined “Full” model including all five TFs. We observed that for all the TF models, the ROC AUC increased dramatically with more stringent p value thresholds and that the XGBoost models consistently predicted the SNP ASE classification better than the GLM and GLMNET models did (Fig 2B). At the most stringent ASE p value threshold (p = 1e-5), all XGBoost models for the TFs independently, as well as for the “Full” model, had similar ROC AUCs (between 0.663 and 0.748) for the cross-validation as well as the test and validation SNP sets (Fig 2C, S3C, S3D, S3 Table). We also investigated the area under the precision-recall curve (AUPRC) and found that, for all three sets of SNPs (cross-validation on the training set, test set and validation set), its fold over baseline was highest for the XGBoost model with p value threshold 1e-5 (S3 Table).

Potential confounding factors associated with chromatin read depth and SNP MAFs could influence the accuracy of ASE prediction. To determine whether the improved performance of XGBoost relative to the linear models was attributable to these factors, we re-trained and evaluated all models using variant subsets stratified by read depth or MAF. For read depth, we divided the training set of variants into ten deciles and independently trained XGBoost, GLM, and GLMNET models within each decile. If the superior performance of XGBoost were primarily driven by read depth, we would expect its performance advantage over the linear models to diminish or disappear at specific read-depth deciles. We performed an analogous analysis for SNP MAFs by dividing the training set of variants into ten MAF deciles and independently training and evaluating each model within each decile. We calculated the ROC AUC for XGBoost, GLM, and GLMNET within each decile. Across the read-depth deciles, XGBoost showed significantly better overall performance than GLM and GLMNET (p = 0.032 and p = 0.043, respectively; paired t-tests; Fig 2D). The same overall pattern was observed across the MAF deciles (p = 0.020 and p = 1.1e-4, respectively; paired t-tests; Fig 2E). These results indicate that the improved predictive performance of XGBoost is not explained by differences in read depth or MAF among variants.

To examine the relative contributions of the different variables to prediction accuracy, we ran XGBoost models using the most stringent ASE p value threshold (p = 1e-5), including only chromatin accessibility data (snATAC-seq read depth, peak score) and SNP MAFs, as well as models including only TF binding data (PWM scores). The XGBoost models, which included only chromatin accessibility data and MAF information, were more accurate at predicting SNP ASE classification than were the models that included only TF binding data (Fig 2F, S3E, S3F, S3 Table). These observations are most likely because each TF recognizes specific sequences and binds only a limited number of genomic positions and therefore helps determine ASE only for a small fraction of heterozygous SNPs.

To investigate whether including additional TFs in the “Full” models would improve predictions, we added twenty TFs that have roles in pancreatic development, pancreatic function and/or diabetes (S2 Table). We observed that increasing the number of TFs from five to twenty-five did not improve the accuracy of the XGBoost or linear models (S2 Fig, S4 Table). This lack of improvement with additional TFs is likely explained by the fact that the PWM scores for any individual TF are informative only for the small fraction of heterozygous SNPs overlapping that TF’s binding sites. Furthermore, many of the 20 additional TFs share similar binding motifs or co-occur at the same regulatory elements as the original five, meaning that their PWM scores provide largely redundant information.

Taken together, our analyses revealed that the XGBoost models performed better than the GLM and GLMNET linear models at predicting SNP ASE classification. The best outcomes were obtained using the most stringent ASE p value threshold (p = 1e-5), indicating that ASE classifications supported by stronger evidence of allelic imbalance were predicted more accurately. Our results suggest that the XGBoost predictions are driven largely by chromatin accessibility variables (snATAC-seq read depth and peak score) and SNP MAF.

### snATAC-seq read depths, peak scores and SNP MAFs are important for predicting outcome

We further examined the variables influencing prediction outcomes in the “Full” XGBoost model (outcome: ASE p-value < 1e-5) via Shapley (SHAP) analysis. We observed that the snATAC-seq read depth feature had the largest SHAP values, indicating that this variable was the most important in the full XGBoost model, followed by SNP MAF, peak score and each TF-associated PWM for both reference and alternate alleles, as well as the delta PWM between reference and alternate alleles (S4 Fig). Several variables had large variability in SHAP values across SNPs, suggesting the presence of nonlinear interactions with other features. Complex nonlinear relationships between the variables and the outcome (predicted SNP ASE) could explain why XGBoost outperforms the linear models. Importantly, the prominence of read depth as a SHAP feature does not imply that the model is learning only detection capability rather than a biological signal. While greater read depth confers greater statistical power for ASE detection, read depth in accessible chromatin regions is itself a biological signal: it reflects the degree of chromatin opening at a regulatory element, which is directly related to transcription factor occupancy and regulatory activity. Consequently, the model’s reliance on read depth is consistent with the biological interpretation that highly active regulatory elements are more likely to harbor functional SNPs with allele-specific effects on TF binding. The correlation between read depth and ASE detectability is therefore both a statistical and biological property of accessible chromatin. This interpretation is supported by the observation that the model trained on chromatin accessibility and MAF information alone substantially outperforms a model trained only on TF binding predictions (Fig 2F), indicating that chromatin accessibility measures capture biologically informative variation beyond mere statistical power.

### UMAP clustering of SHAP values uncovers nonlinear interactions between SNP MAFs and HNF4A PWM scores

To further investigate the associations between the variables and outcomes in the Full XGBoost model, we performed UMAP clustering of all the SNPs on the basis of their SHAP values associated with each variable (Fig 3A). We investigated the properties of the SNPs in the seven UMAP clusters, including 1) the total number of SNPs in each cluster (Fig 3B), 2) the fraction of SNPs with ASE (Fig 3C), 3) their normalized read depth (Fig 3D), 4) their MAF distributions (Fig 3E), and 5) overlap with 18 ChromHMM chromatin states in seven EpiMap pancreatic tissues [36,37] (Fig 3F, S5 Fig).

**Fig 3:**
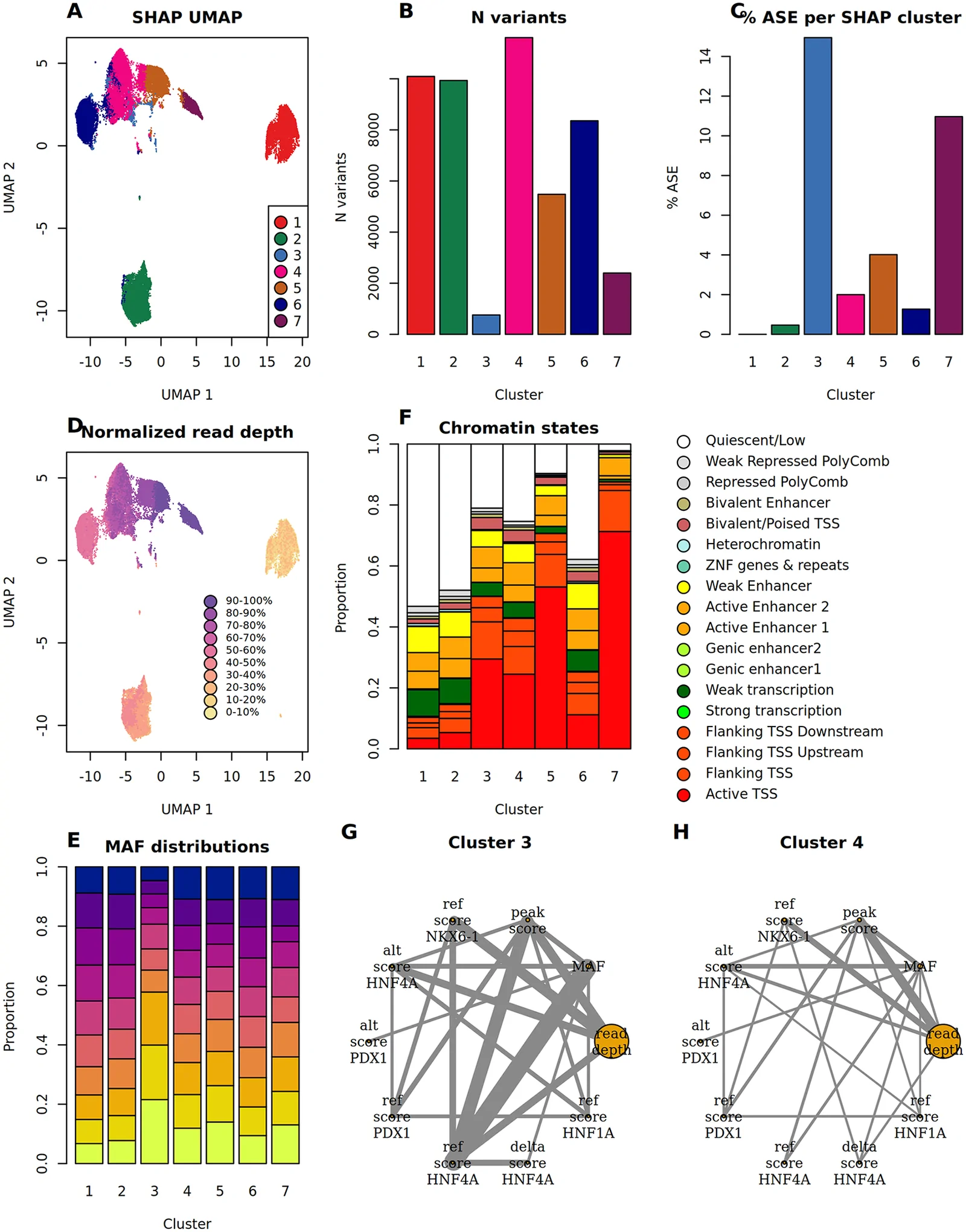
SHAP analysis of the XGBoost model. A) Scatterplot showing the UMAP coordinates of each SNP. UMAP coordinates were calculated from the SHAP values for the model features. The colors represent seven clusters obtained via k-means clustering. The cluster numbers are labeled in the figure. B-C) Bar plots showing B) the number of SNPs per cluster and C) the percentage of SNPs with significant ASE (P value < 1e-5) in each cluster. D) Scatterplot showing the UMAP coordinates of each SNP. The colors represent the read depth deciles. E) Bar plot showing the distribution of MAF deciles among SNPs within the seven clusters. The colors represent the MAF deciles, with lighter colors indicating relatively lower-MAF variants within the analyzed dataset and darker colors indicating relatively higher-MAF variants. All analyzed variants had MAF ≥ 5%. F) Bar plot showing the distribution of overlaps between each SNP and ChromHMM chromatin states in the seven clusters. G-H) Plots showing the SHAP interactions among the ten variables with the strongest effects on model predictions: G) cluster 3; H) cluster 4. Node size is proportional to feature importance, and edge width is proportional to the SHAP interaction value between each pair of features. The SHAP interactions for clusters 1, 2, 5, 6, and 7 are shown in Fig S6.

We observed that the variants in the clusters presented distinct read depth associations, with clusters 1 and 2 accounting for the vast majority of the SNPs in the bottom four deciles (Fig 3B, 3D). Clusters 1 and 2 had a relatively large number of variants, but most did not display ASE (cluster 1: 10,091 SNPs, no ASE SNPs; and cluster 2: 9,933 SNPs, 0.46% ASE SNPs) and were associated with the largest proportion of repressed and quiescent chromatin (56.5% and 51.0%, respectively) and the smallest proportion of promoters (10.3% and 14.6%, respectively). These data indicate that the SNPs in clusters 1 and 2 did not display ASE because they are present in regions of the genome that are repressed (not functionally active) in pancreatic progenitor cells. The SNPs in cluster 6 had similar properties to those in clusters 1 and 2 and had relatively low read depths, a low proportion displaying ASE and a high proportion in quiescent chromatin.

Cluster 7 accounted for the vast majority of SNPs in the highest read-depth decile (Fig 3D). Although cluster 7 contained relatively few SNPs overall, it had a high proportion of SNPs exhibiting ASE (2,398 SNPs, 11.0% ASE; Fig 3C). The vast majority of SNPs in cluster 7 (87.6%) overlapped active transcription start sites (TSSs; 71.2%) or regions flanking TSSs, whereas only 2.44% overlapped repressed or quiescent regions, indicating that this cluster is predominantly composed of variants in active promoter regions. The enrichment of ASE in cluster 7 could reflect both technical and biological factors. Active promoters are highly accessible and therefore tend to have greater chromatin read depth, increasing statistical power to detect allelic imbalance. At the same time, variants within active promoters may have greater potential to alter transcription factor binding or other regulatory processes involved in transcription initiation, resulting in genuine allelic effects. Because promoter accessibility and read depth are correlated, we cannot fully distinguish the contribution of increased statistical detection power from that of larger biological effect sizes to the elevated ASE rate observed in cluster 7. We therefore interpret the enrichment of ASE in cluster 7 as consistent with, but not by itself evidence for, an increased functional impact of variants within active promoter regions (Fig 3F; S5).

Despite having indistinguishable SHAP values for read depth (Bonferroni-adjusted p = 1, t-test; S5–S6 Tables) and similar enrichments across chromatin states, SNPs in cluster 3 were 7.48-fold more likely to exhibit ASE than SNPs in cluster 4 (756 SNPs in cluster 3, 15.0% ASE; Fig 3A–3F). These findings suggest that differences in read depth are unlikely to account for the markedly different proportions of ASE SNPs between these two clusters. To investigate features that might distinguish cluster 3 from cluster 4 and the other clusters, we examined SHAP feature interactions across all clusters. With the exception of cluster 3, the clusters exhibited similar patterns of SHAP feature interactions, with the strongest interaction occurring between read depth and peak score, followed by the interaction between read depth and the reference-allele PWM score for NKX6–1 (Fig 3G–3H; S6 Fig). In contrast, cluster 3 exhibited a distinct pattern characterized by strong interactions involving HNF4A motif scores. The strongest interactions were between (1) MAF and the reference-allele PWM score for HNF4A, (2) peak score and the reference-allele PWM score for HNF4A, (3) read depth and the alternate-allele PWM score for HNF4A, (4) read depth and the reference-allele PWM score for HNF4A, and (5) the reference-allele PWM scores for NKX6–1 and HNF4A (Fig 3G). Notably, SNPs in cluster 3 had significantly lower MAFs than SNPs in all other clusters (Bonferroni-adjusted p = 1.3e-40 to 1.0e-17, t-tests; Fig 3E; S5 Table). Here and throughout the manuscript, “lower-MAF” denotes a relative difference among the analyzed variants, all of which had MAF ≥ 5%; it does not indicate rare or low-frequency variants in the population-genetic sense. These unique interactions in cluster 3 indicate that the SNPs within the cluster are enriched for variants predicted to affect HNF4A binding motifs. The ability to identify these lower-MAF SNPs depends on the peak quality and read depth of the regulatory element, suggesting that their individual effects on chromatin accessibility are likely modest and detectable only within highly accessible elements (Fig 3A–D, F).

Given the unique properties of cluster 3 (the HNF4A/lower-MAF cluster), we further investigated whether its features could be explained by associations among MAF, read depth, and peak score. First, we performed logistic regression to test whether cluster 3 membership (the binary outcome) was associated with MAF. As expected, we observed a strong association between MAF and cluster 3 membership (estimate = −0.620 ± 0.049; p = 1.92e-36). We next included read depth and peak score in the model. MAF remained strongly associated with cluster 3 membership (estimate = -0.613 ± 0.049; p = 1.22e-35), with the magnitude of the MAF coefficient essentially unchanged after adjustment. Read depth (estimate = -0.231 ± 0.035; p = 6.95e-11) and peak score (estimate = -0.129 ± 0.045; p = 4.30e-3) were also independently associated with cluster 3 membership. These results indicate that the association between lower MAF and cluster 3 membership is not explained by its relationships with read depth or peak score.

Because variant MAF and its SHAP contribution are inputs to the UMAP embedding, the identification of a lower-MAF cluster is expected, and excluding MAF and its SHAP contribution from the embedding dissolves cluster 3, as expected for a group defined in part by these features. Thus, the lower MAF of cluster 3 is not itself the biologically informative finding. Rather, what distinguishes this cluster is that these lower-MAF SNPs have a substantially higher proportion of ASE and exhibit a unique pattern of SHAP feature interactions involving HNF4A binding motifs. Together, these findings identify a distinct subset of lower-MAF variants in which HNF4A-related regulatory features are associated with an increased likelihood of allelic effects on chromatin accessibility.

Having determined that MAF remained strongly associated with cluster 3 membership after adjusting for read depth and peak score, we next tested whether the distinctive association of cluster 3 variants with HNF4A binding sites was also associated with greater predicted variant deleteriousness. We used linear regression to test the interaction between cluster 3 membership and HNF4A binding-disruption SHAP scores in predicting Combined Annotation Dependent Depletion (CADD) PHRED scores [40], while including the corresponding scores for the other transcription factors as covariates. Cluster 3 membership alone was not associated with differences in CADD PHRED scores. In contrast, both the HNF4A SHAP score and the interaction between HNF4A SHAP score and cluster 3 membership were significantly associated with higher CADD PHRED scores (estimate = 1.56 ± 0.31, p = 6.05e-7; and estimate = 6.30 ± 2.43, p = 9.49e-3, respectively). Thus, although variants in cluster 3 were not generally predicted to be more deleterious, the relationship between the HNF4A binding-disruption SHAP score and predicted deleteriousness was substantially stronger for variants in cluster 3 than for variants in the other clusters. Specifically, the relationship between HNF4A binding disruption and CADD PHRED score was approximately fivefold steeper in cluster 3. These findings demonstrate that increases in HNF4A binding-disruption SHAP scores were associated with larger increases in CADD PHRED scores for variants in cluster 3 compared with variants in the other clusters.

Taken together, these analyses identified model-predicted nonlinear interactions in cluster 3 among read depth, SNP MAF, peak score, and HNF4A PWM score variables. Because both MAF and its SHAP contribution were included in the UMAP embedding cluster 3’s lower MAF is not an unexpected finding, however, the distinctive interaction structure of cluster 3 is not a direct restatement of the marginal MAF input. Overall, our analyses suggest that, among the variants analyzed in this study, lower-MAF SNPs are more likely to exhibit strong predicted interactions involving HNF4A binding-motif scores than binding-motif scores for the other four TFs.

## Discussion

Our study demonstrates that relatively small snATAC-seq datasets can be effectively used to predict the functional impact of regulatory variants in pancreatic progenitor cells. We show that XGBoost outperforms linear approaches in predicting allele-specific chromatin accessibility because it can capture complex nonlinear interactions among variables. Although alternative nonlinear models, such as deep neural networks, could be applied to this problem, XGBoost provides a combination of nonlinear modeling capacity and interpretable feature contributions through SHAP values. This enables us to investigate the features and interactions underlying regulatory SNP effects rather than focusing solely on improvements in predictive performance.

Importantly, we found that chromatin accessibility and variant context, rather than transcription factor binding predictions alone, are strong predictors of ASE classification. SHAP-based feature importance and clustering revealed distinct regulatory environments among variants with similar patterns of model-predicted effects. For example, cluster 7 was strongly enriched for active promoter regions, whereas other clusters were associated with more quiescent chromatin states. Cluster 3, which had the largest proportion of ASE variants, was distinguished by lower-MAF variants and a unique pattern of predicted interactions involving HNF4A PWM scores, MAF, read depth, and peak score. Although the lower-MAF identity of cluster 3 is not unexpected because MAF and its SHAP contribution were included in the UMAP embedding, its distinctive pattern of predicted interactions involving HNF4A binding-motif scores is not a direct restatement of the marginal MAF input.

HNF4A is a highly conserved pioneer transcription factor with important roles in transcriptional regulation and pancreatic development [41]. Our results suggest that lower-MAF SNPs predicted to affect HNF4A binding are preferentially observed in highly accessible regulatory elements with sufficient read depth and peak quality to detect their allelic effects. The dependence on high read depth and peak scores is consistent with effects on chromatin accessibility that are modest and most readily detectable within highly accessible elements. However, because greater read depth reflects biological accessibility but also increases statistical power to detect ASE, we cannot determine the extent to which this pattern reflects biological context, detection sensitivity, or both. Previous studies have shown that variants within HNF4A binding sites, particularly in accessible chromatin regions, can have pronounced effects on transcriptional regulation [42,43]. Our results extend these observations by identifying a distinctive relationship among allele frequency, chromatin accessibility, and predicted HNF4A binding effects. The association between HNF4A binding-disruption SHAP scores and CADD PHRED scores was stronger for variants in cluster 3 than for variants in the other clusters, suggesting that cluster 3 variants with higher HNF4A SHAP scores may have meaningful functional consequences.

The novelty of our XGBoost-SHAP framework lies in applying an interpretable gradient-boosted ensemble method to the prediction of allele-specific chromatin accessibility from single-nucleus epigenomic data in a cell type-specific developmental context. Deep learning has previously been applied to genomic variant-prioritization tasks [19,20,23], and SHAP-based interpretation has been used to explain genomic machine-learning predictions [17,44]. Here, we integrate snATAC-seq read features, variant MAF, and TF-binding predictions to jointly model ASE outcomes and then use SHAP-value UMAP clustering to identify groups of variants characterized by distinct combinations and interactions of regulatory features. Unlike sequence-to-function models such as Enformer or DNABERT, which operate primarily from sequence and generally depend on large training datasets, our approach incorporates cell type-specific chromatin accessibility measurements directly as biological evidence. This makes it possible to examine regulatory relationships that are specific to pancreatic progenitor cells and is consistent with recent efforts to develop interpretable, biologically grounded machine-learning approaches in functional genomics [17,20].

A key strength of the XGBoost-SHAP framework is its potential to translate model predictions into experimentally testable hypotheses [19,20]. SHAP values provide per-SNP, per-feature measures of each feature’s contribution to model predictions, allowing variants to be prioritized according to the regulatory features driving their predicted ASE effects. SHAP interaction values further identify pairs of features that jointly influence predictions and can therefore reveal nonlinear predictive relationships that would not be apparent from individual feature effects alone. In cluster 3, for example, the strong predicted interactions between HNF4A PWM scores and MAF, read depth, peak score, and NKX6–1 PWM scores identify a specific regulatory context associated with the cluster’s high proportion of ASE variants.

The lower-MAF variants in cluster 3 with strong predicted effects involving HNF4A therefore provide candidates for experimental validation. These variants could be evaluated using allele-specific reporter assays in pancreatic progenitor cells, electrophoretic mobility shift assays to quantify differential HNF4A binding between reference and alternate alleles, or CRISPR base editing in iPSC-derived pancreatic progenitor cells or organoids to directly assess the effects of individual variants on local chromatin accessibility and downstream gene expression. Such experiments would provide a direct test of the regulatory relationships identified by the XGBoost-SHAP framework and could establish which computationally prioritized variants have measurable effects on HNF4A binding and chromatin accessibility. More broadly, integrating cell type-specific snATAC-seq measurements with interpretable machine learning provides a scalable strategy for identifying and prioritizing noncoding variants with potential functional relevance to pancreatic development and disease.

## Limitations of the study

Several limitations of this study should be noted. First, our snATAC-seq cohort comprised only seven iPSC-derived samples, limiting statistical power to detect regulatory events with smaller effect sizes and precluding a comprehensive assessment of interindividual variability in chromatin accessibility. Larger cohorts with matched genotype data will be important for strengthening allele-specific effect estimates and assessing their reproducibility across individuals. Second, our analysis is specific to the iPSC-derived pancreatic progenitor cell (iPSC-PPC) developmental stage. Regulatory patterns, including the lower-MAF signature associated with HNF4A binding sites, may differ in mature beta cells, exocrine cells, or other stages of pancreatic development, and generalization to these cellular contexts will require further investigation. Third, our selection of TFs (HNF4A, HNF1A, NEUROD1, NKX6–1, and PDX1) was guided by their biological importance and data availability. This focused set does not capture the full transcriptional regulatory landscape of pancreatic progenitor cells, and future studies should extend the framework to additional factors as high-quality TF-binding datasets become available. Fourth, the XGBoost model was trained using the iPSCORE population, which may limit its generalizability to populations with different allele-frequency spectra. Validation in genetically diverse populations will therefore be important for establishing the broader applicability of the model. Fifth, SHAP values quantify feature contributions to model predictions and do not constitute direct evidence of biological causality. Experimental validation of SHAP-prioritized variants will be required to establish their functional effects and the regulatory mechanisms underlying the observed associations. Sixth, chromatin read depth is both a biological measure related to chromatin accessibility and a determinant of statistical power in the binomial ASE test. Consequently, our analyses cannot fully separate the biological contribution of accessibility from the increased ability to detect ASE at greater read depths. However, when variants were stratified into read-depth deciles, XGBoost showed significantly better overall predictive performance across deciles than the linear models. Thus, although read depth contributes to ASE detection, differences in read depth alone do not explain the superior predictive performance of XGBoost. Seventh, because variant MAF and its SHAP contribution were inputs to the UMAP embedding, the identification of a lower-MAF cluster is expected and is not independent of the features used to construct the embedding. Consistent with this, cluster 3 was no longer detected when MAF and its SHAP contribution were excluded from the embedding. The more specific and biologically informative observation is therefore not cluster 3’s lower-MAF identity itself, but its high ASE enrichment and distinctive pattern of predicted interactions involving HNF4A binding-motif scores. This pattern of predicted HNF4A interactions is not a direct restatement of the marginal MAF input. Finally, although the data were generated using snATAC-seq, ASE was assessed using reads aggregated across all nuclei, which were predominantly NKX6–1 + pancreatic progenitors, rather than at single-cell resolution. The resulting framework should therefore be considered cell-population-specific rather than fully single-cell-resolved. Future studies with larger datasets and sufficient per-cell allelic coverage may enable these regulatory effects to be examined at true single-cell resolution.

## Conclusion

This work demonstrates the technical feasibility and biological utility of integrating snATAC-seq with computational modeling and an interpretable XGBoost-SHAP framework to functionally annotate noncoding variants within a specific developmental context. Our framework contributes to the growing toolkit for functional annotation of noncoding variation and highlights the value of integrating cell type-specific epigenomic measurements with computational modeling to advance precision genomics. By incorporating direct measurements of chromatin accessibility and regulatory context, this approach provides a distinct and potentially complementary strategy to sequence-based foundation models such as Enformer and DNABERT. Ultimately, this work provides a foundation for extending predictive approaches across the noncoding genome, with the long-term goal of predicting the functional consequences of variants regardless of their genomic location.

## Supporting information

S1 Text PPC differentiation methods. This text describes how seven iPSC lines from unrelated iPSCORE donors were identified, how matching whole-genome sequencing from each donor’s blood was generated and processed, and the methods to differentiate PPCs from iPSCs.
(DOCX)

S1 Fig iPSC-PPCs are largely composed of NKX6–1 + progenitors. (A) UMAP plot of snATAC-seq data from single nuclei from 7 iPSC-PPC samples. Each point represents a single nucleus color-coded by its assigned cluster. (B) Stacked bar plot showing the fraction of cells from each sample assigned to each cluster in snATAC-seq. The color coding corresponds to the clusters in panel A. (C) Heatmap comparing the z-normalized motif activity scores from chromVAR of pancreatic-associated transcription factors. Each score is then compared to its normalized gene expression in scRNA-seq clusters [6] labeled in gray. We integrated the snATAC-seq samples via Seurat [45], and although five clusters were observed, the vast majority of the nuclei (24,068 nuclei, 92.5% of the 26,026 passing all the quality filters) belonged to a single cluster. We examined the activity of transcription factors associated with pancreatic development via chromVAR [13] and compared their correspondence with gene expression from scRNA-seq data previously generated from these same samples [6]. We observed similar iPSC-PPC cell type populations between the transcriptomic and epigenomic analyses. The mesendoderm cluster expressed markers for early embryonic development, including transcription factors related to the TFAP family [46]. The most prevalent cluster corresponded to pancreatic progenitor cells (NKX6–1 + progenitors). In addition to NKX6–1 + progenitors, we also identified three small clusters at later developmental stages: early endocrine cells, which are associated with high motif activities of SOX4, SOX10 and SOX13 [47]; endocrine cells, which present high motif activities of NKX2–2 and NEUROD1 [48]; and precursors for ductal cells (ETV1, ETS1 and ETS2; referred to as early ductal cells) [49]. Overall, our results show that NKX6–1 + progenitors are the predominant cell type captured by snATAC-seq.
(GIF)

S2 Fig Comparing models using five and 25 TFs. Scatterplot showing a comparison of the ROC AUC between the full models including snATAC-seq accessibility (read depth and peak score) and MAF features and PWMs for the five TFs described in the main text, and the models built using the PWMs of 20 additional TFs together with the other model features. S2 Table lists the pancreatic development-related functions of all 25 TFs. The figure shows that, across model types and ASE p value thresholds, adding more TFs did not substantially change ROC AUC. The ROC AUCs shown are for the test set.
(TIF)

S3 Fig Model predictive performance in the test and validation sets. A-D) ROC curves for A, B) predictions using only PDX1 at multiple ASE p value thresholds and models and C, D) XGBoost models including each transcription factor at an ASE p value threshold of 1e-5. A, C) Test set; B, D) validation set. E-F) ROC curves for models built using only transcription factor information (solid lines) or ATAC-seq accessibility (read depth, peak score) and MAF (dashed lines) in the E) test set and F) validation set. In the panels, “Sequence” refers to these accessibility and frequency features rather than the underlying DNA sequence. “Full model” indicates the model that uses all five TFs.
(TIF)

S4 Fig SHAP importance for the Full XGBoost model. SHAP importance plot: The input variables were ranked from top to bottom according to their mean absolute SHAP values for the ASE predictions. The colors represent the feature value of each SNP for the indicated variable. The plot was generated via the function sv_importance (kind = “beeswarm”) from the shapviz package in R.
(TIF)

S5 Fig Chromatin state distributions in each cluster. Bar plots showing the distribution of overlaps of each SNP with ChromHMM chromatin states for each of the seven EpiMap pancreatic tissues.
(TIF)

S6 Fig SHAP interactions within each cluster. Plots showing the SHAP interactions between the ten variables that have the strongest effect on the model in clusters 1, 2, 5, 6 and 7 (Fig 3). The interactions were calculated via the shap.prep.interaction function in SHAPforxgboost 0.1.3 in R. Node size is proportional to feature importance, whereas edge width is proportional to the interaction value between each pair of features. We used SHAP interaction values to examine whether the predictive influence of certain features depended on other features.
(PNG)

S1 Table snATAC-seq statistics. snATAC-seq statistics for each sample are provided, including sequencing depth, number of nuclei and number of reads per nucleus across two snATAC-seq pooled samples (ATAC_Pool_1 and ATAC_Pool_2). We also provide the number of nuclei and the number of fragments per sample.
(XLSX)

S2 Table Description of TF function in PPC and pancreatic metabolism. For each of the 25 TFs used in the prediction models, we provide a description of the rationale regarding why it was selected. All the TFs are involved in pancreatic development, function and/or diabetes.
(XLSX)

S3 Table Model accuracies. Prediction accuracies of the three models (GLM, GLMNET and XGBoost) described in the text are shown for each of the five transcription factors individually, as well as all five combined (i.e., the “Full” model), for each ASE p value threshold (0.05, 0.01, 0.001 and 1e-5), test type (variables included) and SNP set analyzed: 1) training: cross-validation on the training set; 2) test set; or 3) validation set. “Test type” indicates whether the model was built using only the sequence information (“only seq”; chromatin accessibility and MAF features), only the TF information (“only TF”) or both (“full”). Accuracies were calculated on the training (cross-validation), test and validation sets. The PPV, NPV, ROC AUC, area under the precision‒recall curve (AUPRC), precision, recall, sensitivity, specificity, F1 statistic and optimal threshold for discriminating between positive and negative predictions are shown.
(XLSX)

S4 Table Model accuracies for the additional TFs. The table is organized as S3 Table and shows the model accuracy associated with each of the 20 TFs used to examine model performance if additional TFs were included. Each of the 20 TFs that were examined independently had similar ROC AUCs as the original five TFs did. The model indicated in the TF column as a combination includes all 25 TFs.
(XLSX)

S5 Table Pairwise comparisons of SHAP feature distributions between clusters. Pairwise comparison of SHAP feature distributions between the seven clusters (Fig 3A). The SHAP features are indicated in the column “variable”. The table provides test results for both the normalized values and the SHAP scores, as indicated in the “score type” column. The mean values for each of the two clusters, as well as the p value (t test) and p value adjusted with Bonferroni’s method, are shown.
(XLSX)

S6 Table Normalized values, SHAP scores and cluster information for all heterozygous variants. For each variant in the training set, the normalized values and SHAP scores of each variable are presented. The cluster (Fig 3A) containing the variant, its UMAP coordinates calculated on SHAP scores, its ASE -log10 (p value) and major allele frequency, and outcome (0 = ASE p value > 1e-5; 1 = ASE p value ≤ 1e-5) are also presented. The first column shows the variant ID, including the UUID for its associated subject [29] and the SNP position.
(TXT)
