# Supplementary material for: XGBoost-SHAP analysis prioritizes lower-frequency variants predicted to affect HNF4A binding motifs in pancreatic progenitors
Source: PLoS Genet. 2026 Nov 4;22(11):e1012361. doi: 10.1371/journal.pgen.1012361 (PMC13754212; doi:10.1371/journal.pgen.1012361)
Supplement: Supplementary file 1 [file pgen.1012361.s001.docx]

# S1 Text

## iPSCORE subject information

We obtained 7 iPSC lines reprogrammed from the fibroblasts of 7 unrelated subjects in the iPSCORE collection [1,2]. Each subject was assigned a Universally Unique Identifier (UUID) and an iPSCORE_ID (e.g., iPSCORE_4_1), which designates a family (4) and an individual (1). From each subject, whole-blood samples were collected and used to generate and process whole-genome sequence (WGS) data as previously described [1,3,4]. Briefly, reads were aligned against the human b37 genome with decoy sequences via BWA-MEM with the default parameters [5]. We applied the GATK best-practices pipeline for variant calling, which includes indel-realignment, base-recalibration, genotyping via HaplotypeCaller, and finally joint genotyping via GenotypeGVCFs [6]. The recruitment of these individuals was approved by the Institutional Review Boards of the University of California, San Diego, and the Salk Institute (project no. 110776ZF).

## iPSC-PPC derivation

We derived pancreatic progenitor cells (PPCs) from the 7 iPSC lines (iPSC-PPCs) via the STEMdiff Pancreatic Progenitor Kit (StemCell Technologies), which was previously described in detail [2]. Each iPSC-PPC differentiation was given a unique differentiation identifier (UDID; PPC_0XX). The names of all the molecular samples generated from that differentiation contain the UDID. We briefly describe the derivation protocol below.

*Expansion of iPSCs:* One vial from each of the 7 iPSC lines was thawed into mTeSR1 medium containing 10 μM ROCK inhibitor (Selleckchem) and plated on one well of a 6-well plate coated with Matrigel. iPSCs were grown until they reached 80% confluency and then passaged using a 2 mg/ml solution of Dispase II (Thermo Fisher Scientific). To obtain a sufficient number of iPSCs for differentiation, iPSCs were passaged twice: 1) cells from the first passage were plated in three wells of a 6-well plate (ratio of 1:3), and 2) cells from the second passage were plated in six wells of a 6-well plate (ratio of 1:2).

*Monolayer plating (day 0; D0)*: When the confluency of the iPSCs in the six wells of a 6-well plate reached 80%, the cells were dissociated into single cells via Accutase (Innovative Cell Technologies Inc.). Single iPSCs were resuspended at a concentration of 1.85 x10^6^ cells/ml in mTeSR containing 10 μM ROCK inhibitor and plated in six wells of a 6-well plate. The cells were grown for approximately 16 to 20 hours to achieve uniform 90–95% confluency (3.7x10^6^ cells/well; approximately 3.9x10^5^ cells/cm^2^).

*Differentiation:* Differentiation of the confluent iPSC monolayers was initiated by the addition of STEMdiff Stage Endoderm Basal medium supplemented with Supplement MR and Supplement CJ (2 ml/well) (D1). All the following media changes were performed every 24 hours following the initiation of differentiation (2 ml/well). On D2 and D3, the medium was changed to fresh STEMdiff Stage Endoderm Basal medium supplemented with Supplement CJ. On D4, the medium was changed to STEMdiff Pancreatic Stage 2–4 Basal medium supplemented with Supplement 2A and Supplement 2B. On D5 and D6, the medium was changed to STEMdiff Pancreatic Stage 2–4 Basal medium supplemented with Supplement 2B. On D7, D8 and D9, the medium was changed to STEMdiff Pancreatic Stage 2–4 Basal medium supplemented with Supplement 3. On D10, D11, D12, D13 and D14, the medium was changed to STEMdiff Pancreatic Stage 2–4 Basal medium supplemented with Supplement 4.

*Harvest:* On D15, the cells were dissociated via Accutase, collected, counted, and either processed fresh (scRNA-seq) or cryopreserved (scRNA-seq and snATAC-seq).

# References

1. Panopoulos AD, D’Antonio M, Benaglio P, Williams R, Hashem SI, Schuldt BM, et al. iPSCORE: A Resource of 222 iPSC Lines Enabling Functional Characterization of Genetic Variation across a Variety of Cell Types. Stem Cell Reports. 2017;8:1086–100. https://doi.org/10.1016/j.stemcr.2017.03.012

2. Nguyen JP, Arthur TD, Fujita K, Salgado BM, Donovan MKR, Matsui H, et al. eQTL mapping in fetal-like pancreatic progenitor cells reveals early developmental insights into diabetes risk. Nat Commun. Nature Publishing Group; 2023;14:6928. https://doi.org/10.1038/s41467-023-42560-4

3. DeBoever C, Li H, Jakubosky D, Benaglio P, Reyna J, Olson KM, et al. Large-Scale Profiling Reveals the Influence of Genetic Variation on Gene Expression in Human Induced Pluripotent Stem Cells. Cell Stem Cell. 2017;20:533-546.e7. https://doi.org/10.1016/j.stem.2017.03.009

4. D’Antonio M, Nguyen JP, Arthur TD, iPSCORE Consortium, Matsui H, D’Antonio-Chronowska A, et al. Fine mapping spatiotemporal mechanisms of genetic variants underlying cardiac traits and disease. Nat Commun. 2023;14:1132. https://doi.org/10.1038/s41467-023-36638-2

5. Li H. Aligning sequence reads, clone sequences and assembly contigs with BWA-MEM [Internet]. arXiv; 2013 [cited 2025 July 13]. https://doi.org/10.48550/arXiv.1303.3997

6. Poplin R, Ruano-Rubio V, DePristo MA, Fennell TJ, Carneiro MO, Auwera GAV der, et al. Scaling accurate genetic variant discovery to tens of thousands of samples [Internet]. bioRxiv; 2018 [cited 2025 July 13]. p. 201178. https://doi.org/10.1101/201178
