# Supplementary material for: XGBoost-SHAP analysis prioritizes lower-frequency variants predicted to affect HNF4A binding motifs in pancreatic progenitors
Source: PLoS Genet. 2026 Nov 4;22(11):e1012361. doi: 10.1371/journal.pgen.1012361 (PMC13754212; doi:10.1371/journal.pgen.1012361)
Supplement: Supplementary file 3 [file pgen.1012361.s016.docx]

# Editor:

We have noticed that you have uploaded Supporting Information files, but you have not included a list of legends. Please add a full list of legends for your Supporting Information files after the references list.

Supplemental figure and table legends are included in the Supplementary Information file. We copied them to the main text after the reference list.

# Reviewer #1

The authors have substantially addressed my concern regarding the potential confounding effect of read depth. I appreciate the addition of the read-depth-stratified analyses, in which XGBoost, GLM, and GLMNET were independently trained and evaluated within read-depth deciles, as well as the corresponding MAF-stratified analysis. One point could still be clarified further. The additional regression analysis shows that the association between MAF and cluster 3 membership remains after adjustment for read depth and peak score, which is informative. However, this does not strictly demonstrate that the cluster itself, or its HNF4A-related interaction pattern, is independent of read depth and peak score. I therefore suggest slightly tempering statements such as “cluster 3’s MAF properties were independent of read depth and peak score” and instead stating more precisely that the association between MAF and cluster 3 membership remained after adjustment for these variables.

Thank you for your comment. We have updated the text as follows:

Having determined that MAF remained strongly associated with cluster 3 membership after adjusting for read depth and peak score, we next tested whether the distinctive association of cluster 3 variants with HNF4A binding sites was also associated with greater predicted variant deleteriousness.

# Reviewer #2

The authors have done a commendable job addressing my comments. They added analyses of model performance across read-depth strata, which were requested independently by the other reviewers and provide greater context for the performance of their approach. They have also tempered the interpretation of the cluster 7 result to note that detection power and biological effect are not separable with the current data, which addresses my concern. The conclusions and phrasing now better reflect the support in the data. I have no further concerns and recommend acceptance.

Thank you.

# Reviewer #3

The authors have addressed my comments

Thank you.
