# Supplementary material for: XGBoost-SHAP analysis prioritizes lower-frequency variants predicted to affect HNF4A binding motifs in pancreatic progenitors
Source: PLoS Genet. 2026 Nov 4;22(11):e1012361. doi: 10.1371/journal.pgen.1012361 (PMC13754212; doi:10.1371/journal.pgen.1012361)
Supplement: Supplementary file 2 [file pgen.1012361.s015.docx]

We thank the Editor and the three reviewers for their careful and constructive evaluation of our manuscript. The reviews converge on a central, important point—the need to distinguish the contribution of read depth as a determinant of statistical detection power from its role as a biological measure of chromatin accessibility—and on the related concern that some of our language implied stronger causal and mechanistic claims than our model-based evidence supports. We have taken these points seriously. In this revision, we have (i) softened causal and “active HNF4A binding” language throughout the manuscript, including the title, Abstract, Results, and Discussion, so that claims are framed as model-based predictions and associations rather than direct evidence of transcription factor binding or selection; (ii) revised the manuscript throughout, including the Abstract, Author Summary, Introduction, Results, Discussion, and Limitations, to clarify that read depth is both a biological measure related to chromatin accessibility and a determinant of statistical power to detect ASE, and that the lower-MAF identity of cluster 3 was expected because MAF and its SHAP contribution were inputs to the UMAP embedding; (iii) clarified the assumptions of our statistical comparisons and the aggregated (cell-population-level) nature of the ASE analysis; and (iv) corrected the figure-reference, citation, terminology, and formatting errors that the reviewers identified.

Below we respond to each point in turn. Reviewer comments are shown in italics; our responses follow. Pertinent manuscript changes are highlighted in the revised manuscript. Because line numbers shift with tracked changes, we refer to sections by name.

# Editor Comments

**Comment 1:** All the referees have raised concerns about the potential confounding between read depth and the ASE label, which may indicate the model performance reflects statistical detection power rather than genuine regulatory biology. The authors should perform additional analysis to convince the reviewers and the editors.

We agree this is the most important issue raised by all three Reviewers (Reviewer 1 comment 1, Reviewer 2 comment 2 and Reviewer 3 comment 2), and we address it in multiple ways:

1. We note the evidence in our current analyses that argues against a purely statistical explanation: a model trained on accessibility features (read depth, peak score) and MAF substantially outperforms a model trained on TF-binding (PWM) features alone (Figure 2F), and cluster 3 and cluster 4 have statistically indistinguishable read-depth SHAP values (Bonferroni-corrected p = 1; Figure 3) yet differ 7.5-fold in ASE rate, a difference that read depth alone cannot explain.
2. We have removed language that asserted read depth reflects biology rather than detection power without direct support, and we now present the dual nature of read depth explicitly and cautiously.
3. While we cannot fully distinguish between the biological and statistical roles of read depth, we performed a read-depth-stratified evaluation, as suggested by Reviewers 1 and 3: we divided variants into read-depth deciles, independently trained and evaluated XGBoost, GLM, and GLMNET within each decile, and compared ROC AUC across deciles. XGBoost showed significantly better overall performance across the read-depth deciles than GLM and GLMNET (paired t-test p = 0.032 and p = 0.043, respectively). We performed an analogous analysis across MAF deciles and observed the same overall pattern (p = 0.020 and p = 1.1e-4, respectively). These results indicate that the improved predictive performance of XGBoost is not explained by differences in read depth or MAF among variants. These results are now shown in Figure 2D–E.
4. Additionally, following Reviewer 1 (comment 1), we tested the effects of read depth and peak score on cluster 3 (the HNF4A/lower-MAF cluster) by performing two logistic regressions with the outcome defined as membership in cluster 3:
   a. First, we simply tested if MAF alone is associated with Cluster 3 assignment using logistic regression. As expected, the association is highly significant (estimate = -0.620, standard error = 0.049, p = 1.92e-36), indicating that variants included in Cluster 3 have lower MAF than variants included in other clusters;
   b. Next, we expanded the logistic regression model by including read depth and peak score. We tested: Cluster 3 ~ MAF + read depth + peak score, and found that all three associations are significant: MAF (estimate = -0.613, standard error = 0.049, p = 1.22e-35); read depth (estimate = -0.231, standard error = 0.035, p = 6.95e-11) and peak score (estimate = -0.129, standard error = 0.045, p = 4.30e-3). These results indicate that adjusting by read depth and peak score does not reduce the effects of MAF on cluster 3.

**Comment 2:** Some causal language throughout the manuscript regarding active HNF4A binding and selective constraint may be too strong, as SHAP interactions reflect model explanations rather than direct evidence of TF binding or function. I would recommend the authors softening the tone.

We have softened this language throughout the manuscript. The title now reads “XGBoost-SHAP analysis prioritizes lower-frequency variants predicted to affect HNF4A binding motifs in pancreatic progenitors.” In the Abstract, Results, Discussion, and Limitations, phrases implying direct HNF4A binding or causality have been replaced with prediction- and association-based wording. We retained “consistent with selective constraint on HNF4A-bound regulatory elements” as a cautious interpretation supported by the orthogonal CADD analysis described below in response to Reviewer 3, Comment 4. We also state in the Introduction and Limitations that SHAP contributions represent predictive associations rather than causal effects and require orthogonal computational support and/or experimental validation before causal interpretation.

# Journal Requirements

**Comment 3:** Please ensure the CRediT author contributions are completed for all co-authors; contributions are required for Matteo D’Antonio.

We have completed the CRediT taxonomy for all co-authors in the submission form.

**Comment 4:** Please provide an Author Summary (150–200 words) between the Abstract and the Introduction.

We have added an Author Summary as a new section between the Keywords/Abstract and the Introduction, written for a general audience.

**Comment 5:** Please remove trademark/copyright symbols (TM on pages 6 and 39).

We have removed the ™ symbol from both instances of “STEMdiff™” (Methods and Supplemental Methods).

**Comment 6:** Your Financial Disclosure lists S10OD026929, but the submission form does not indicate it.

Thank you for catching this discrepancy. The Financial Disclosure in the manuscript is correct and complete; we have updated the submission form so that grant S10OD026929 (NIH SIG; UC San Diego IGM Genomics Center NovaSeq 6000) is included, matching the manuscript. We confirm that the funders had no role in study design, data collection and analysis, decision to publish, or preparation of the manuscript.

**Comment 7:** We have amended your Competing Interest statement to comply with journal style; please confirm it is correct.

We have reviewed the amended Competing Interests statement and confirm that it is correct. The authors declare no competing interests.

# Reviewer #1

**Comment 1:** Read depth confounds interpretation of performance at stringent threshold. The strongest predictor is read depth… variants with higher read depth are mechanically more likely to achieve stringent significance even for the same allelic effect size… As a sensitivity analysis, the authors could use beta-binomial or allelic ratio models with read depth modeled explicitly, downsample high-depth variants to a common depth, or repeat the analysis within read-depth/peak-score-matched strata. It would also be important to show whether the HNF4A cluster persists when read depth and peak score are removed, residualized, or matched across clusters.

We agree with the reviewer that read depth increases the power of the binomial ASE test, so that detectability and read depth are coupled at stringent thresholds (see Editor comment 1, Reviewer 2 comment 2, and Reviewer 3 comment 2). To address this concern, we divided variants into read-depth deciles, independently trained and evaluated XGBoost, GLM, and GLMNET within each decile, and compared ROC AUC across deciles. XGBoost showed significantly better overall performance across the read-depth deciles than GLM and GLMNET (paired t-test p = 0.032 and p = 0.043, respectively). An analogous analysis across MAF deciles showed the same overall pattern (p = 0.020 and p = 1.1e-4, respectively). These results indicate that differences in read depth or MAF alone do not explain XGBoost’s overall predictive advantage across deciles (Figure 2D–E).

To further address this concern, we have removed the assertion that read depth reflects biology “rather than” detection power and now state explicitly that read depth may have both a biological and statistical role. Two pieces of existing evidence indicate the model is not learning detection power alone: (i) clusters 3 and 4 are statistically indistinguishable in read-depth SHAP values (Bonferroni-corrected p = 1) yet differ 7.5-fold in ASE rate, so a variable beyond read depth must drive the difference; and (ii) accessibility-plus-MAF models outperform TF-only models, showing accessibility features carry information beyond a null.

To test the effects of read depth and peak score on cluster 3 (the HNF4A/lower-MAF cluster), we performed two logistic regressions with the outcome defined as membership in cluster 3:

1) Cluster 3 ~ MAF: the association is, as expected, highly significant (estimate = -0.620, standard error = 0.049, p = 1.92e-36)
2) Cluster 3 ~ MAF + read depth + peak score: all three associations are significant:
   a. MAF: estimate = -0.613, standard error = 0.049, p = 1.22e-35
   b. read depth: estimate = -0.231, standard error = 0.035, p = 6.95e-11
   c. peak score: estimate = -0.129, standard error = 0.045, p = 4.30e-3

These results indicate that the association between lower MAF and cluster 3 membership is not explained by its relationships with read depth or peak score. We have added this analysis to the text.

**Comment 2:** The low-MAF cluster is not an independent discovery because MAF is a model input. Since MAF is included as a model input and SHAP values for all features are used to construct the UMAP and clusters, the observation that one SHAP-defined cluster is enriched for low-MAF variants is not an independent discovery. The authors should clarify whether the low-MAF cluster persists when MAF and MAF-SHAP features are excluded from the UMAP + clustering.

This is an important point. We agree that because MAF and its SHAP contribution were inputs to the UMAP embedding, the lower-MAF identity of cluster 3 was expected and is not, by itself, an independent biological discovery. We now state this explicitly in the Results, Discussion, and Limitations. The biologically informative findings are cluster 3’s high ASE enrichment and its distinctive pattern of predicted interactions involving HNF4A binding-motif scores.

We also performed the reviewer’s requested sensitivity analysis. When MAF and its SHAP contribution were excluded from the UMAP embedding and the SNPs were re-clustered, cluster 3 no longer formed as a discrete cluster (data not shown). This result confirms that the cluster’s lower-MAF identity depends on the features used to construct the embedding, as expected for a group defined in part by MAF and its SHAP contribution. We therefore do not interpret the existence of a lower-MAF cluster as an independent discovery.

The substantive finding is more specific: among the variants analyzed in this study, lower-MAF SNPs were more likely to exhibit strong predicted interactions involving HNF4A binding-motif scores than binding-motif scores for the other four TFs. This HNF4A-related pattern is not a direct restatement of the marginal MAF input. Moreover, cluster 3 had high ASE enrichment, and CADD analyses supported a stronger relationship between HNF4A binding-disruption SHAP scores and predicted deleteriousness in cluster 3. The CADD analysis is described below in response to Reviewer 3, Comment 4. We revised the manuscript to distinguish these biologically informative findings from the expected lower-MAF identity of the cluster.

**Comment 3:** SHAP interactions are not direct evidence of HNF4A binding. SHAP interactions are explanations of a trained predictive model, not direct evidence of TF binding or functional impact… If such data are not available, the conclusion should be softened to “variants predicted to affect HNF4A motifs” rather than “active HNF4A binding sites.”

As requested by the reviewer, we have softened this conclusion throughout the manuscript, including the title. We now refer to “variants predicted to affect HNF4A motifs/binding” rather than “active HNF4A binding sites,” and we describe SHAP results as predictive associations rather than direct evidence of TF binding or causality. We agree that direct experimental validation would strengthen the claim. The Discussion and Limitations now emphasize the need for follow-up experimental validation. Our FIMO-based PWM analysis quantifies predicted motif disruption (REF versus ALT PWM scores), which is the basis for the softened “predicted to affect motifs” language.

**Comment 4:** Overstated biological certainty in title, abstract, conclusion, discussion. Several statements imply the model reveals functional variant effects, selective constraints, or mechanisms of MODY1 phenotypic variability… I would suggest revising the title and abstract to use more cautious wording.

We have revised the title and Abstract to use more cautious, prediction-based wording, consistent with the reviewer’s suggested framing, and softened “selective constraint on HNF4A binding” to “consistent with selective constraint on HNF4A-bound regulatory elements.” This interpretation is supported by the orthogonal CADD analysis described below in response to Reviewer 3, Comment 4. We retain the MODY1 context as a motivated hypothesis rather than a demonstrated mechanism, and the manuscript now states that SHAP-based prioritization generates hypotheses that require orthogonal computational support and/or experimental validation.

## Reviewer #1 — Minor points

**Comment 5:** Page 5: “cell type-specific” analysis was not well-established; despite snATAC-seq + clustering, the main analysis was still cell-type-agnostic.

We agree. Because ASE was computed from reads aggregated across the predominant progenitor population rather than at single-cell resolution, we have added a limitation stating that the framework is best described as cell-population-specific rather than fully cell-type-resolved, and we have tempered the corresponding claims.

**Comment 6:** Figure 1 caption defines ASE as allele-specific expression, but the main text defines it as allele-specific effect (and it is chromatin accessibility, not expression).

Corrected. The Figure 1 caption now reads “allele-specific effects (ASE) on chromatin accessibility,” consistent with the main text.

**Comment 7:** In the section on UMAP clustering, Figure 2 was referred to where Figure 3 was intended.

Corrected. All erroneous “Figure 2” references in that Results paragraph (panels A–D, E, F, G–H) now refer to Figure 3, matching the figure and its legend.

# Reviewer #2

**Comment 1:** The introduction statement “High read depth and peak scores are linked to SNPs in highly active functional elements, which are more likely to show ASE” is circular; it would be more stable simplified to “SNPs in putative functional elements with high read depth and peak scores are more likely to show ASE.”

We have adopted the reviewer’s exact rewording, removing the circular phrasing.

**Comment 2:** It isn’t clear how sensitive the model is to sequencing depth or total number of cells… read depth is inherently linked to the binomial test’s power… the promoter/Cluster 7 argument is dismissed with an assertion not backed by data… the one cluster defined by highest read depth is most exposed to detection bias… an analysis that distinguishes the two interpretations (matched-depth effect sizes) would strengthen the paper.

We agree and have removed the unsupported assertion about cluster 7. The cluster 7 paragraph now states that promoter accessibility and read depth are correlated and that we cannot fully distinguish the contribution of increased statistical detection power from that of larger biological effect sizes. We therefore interpret the ASE enrichment as consistent with, but not by itself evidence for, an increased functional impact of variants within active promoter regions. To address the broader depth concern, we divided variants into read-depth deciles, independently trained and evaluated XGBoost, GLM, and GLMNET within each decile, and compared ROC AUC across deciles. XGBoost showed significantly better overall performance across the read-depth deciles than GLM and GLMNET (paired t-test p = 0.032 and p = 0.043, respectively). The analogous MAF-decile analysis showed the same overall pattern (p = 0.020 and p = 1.1e-4, respectively). These results indicate that the improved predictive performance of XGBoost is not explained by differences in read depth or MAF among variants (Figure 2D–E).

**Comment 3:** The conclusions focus on the HNF4A association, but the paper is largely about building a model to predict functional variants. Do the authors want to make any conclusions about the method itself?

We agree that the methodological contribution deserves emphasis. We now highlight throughout the Abstract, Author Summary, Introduction, Discussion, and Conclusion that the primary contribution is an interpretable XGBoost-SHAP framework that integrates single-nucleus chromatin accessibility, variant MAF, and TF-binding predictions; captures nonlinear predictive relationships; and uses per-variant SHAP values and SHAP interactions to generate experimentally testable hypotheses. The HNF4A/lower-MAF observation is presented as an illustrative application of this framework rather than as its sole conclusion.

Comment 4: AUPRC is only reported in Supplemental Table 3… ROC AUC stays high under imbalance… reporting AUPRC as fold-over-baseline when performance is claimed would clarify the magnitude of improvement.

We appreciate this suggestion, which we agree improves interpretability under class imbalance. AUPRC for all models and thresholds is reported in Tables S3 and S4; the baseline AUPRC equals the positive-class prevalence at each threshold. We now report the XGBoost AUPRC as a fold-over-baseline where headline performance is claimed. We show that, for the full model, AUPRC-over-baseline is highest with XGBoost at the most stringent p = 1e-5 threshold. We have added AUPRC-over-baseline to Tables S3 and S4 and describe AUPRC in the Results section, “XGBoost models predict ASE outcomes better than linear models do.”

**Comment 5:** In the HNF4A discussion, the authors state the low-frequency variants in cluster 3 have “modest impact.” Do they mean modest population burden or modest impact on ASE? The next sentence says variants in the binding sites have pronounced effects on transcriptional regulation — please clarify.

We have clarified this. The Discussion now states that the dependence on high read depth and peak scores is consistent with effects on chromatin accessibility that are modest and most readily detectable within highly accessible elements. It also explicitly acknowledges that read depth reflects biological accessibility but also increases statistical power to detect ASE, and that these roles cannot be fully distinguished here. We distinguish this interpretation from prior reports that particular variants within HNF4A binding sites can have pronounced effects on transcriptional regulation.

**Comment 6:** For Figure 2D, mention in the legend that “sequence only” means the read depth, peak score, and MAF data, not the DNA sequence.

Corrected. The Figure 2F legend now states that, in the panel, “Sequence” refers to chromatin-accessibility features (snATAC-seq read depth and peak score) and SNP MAF rather than the underlying DNA sequence.

# Reviewer #3

## Major points

**Comment 1:** I’m confused about the “low frequency” claims, since the authors already filtered MAF > 5%, which is the definition of common variants.

We thank the reviewer for pointing out the confusion over our use of “low-frequency.” All analyzed variants had MAF ≥5%. We therefore use “lower-MAF” to describe a relative difference among the analyzed variants; it does not indicate rare or low-frequency variants in the population-genetic sense. We now explicitly state that all analyzed variants had MAF ≥5% in two places in the Methods, in the Results, and in the Figure 3 legend. We also clarify the relative lower-MAF interpretation in the Abstract, Author Summary, Discussion, and Limitations and use “lower-MAF” consistently when describing cluster 3. The title retains the broader phrase “lower-frequency variants,” while the manuscript makes its relative meaning explicit.

**Comment 2:** Recommend a sensitivity check controlling read depth: bin SNPs into read-depth deciles and recompute ROC AUC and AUPRC within each stratum. If within-stratum AUC remains above 0.5 and XGBoost still beats linear models, the biological signal is supported; otherwise interpret with caution.

We agree this is the decisive test and have adopted it as the primary sensitivity analysis, consistent with the Editor’s request and Reviewers 1 and 2 (see Editor comment 1, Reviewer 1 comment 1, and Reviewer 2 comment 2). We divided variants into read-depth deciles, independently trained and evaluated XGBoost, GLM, and GLMNET within each decile, and compared ROC AUC across deciles. XGBoost showed significantly better overall performance across the read-depth deciles than GLM and GLMNET (paired t-test p = 0.032 and p = 0.043, respectively). An analogous analysis across MAF deciles showed the same overall pattern (p = 0.020 and p = 1.1e-4, respectively). These results indicate that the improved predictive performance of XGBoost is not explained by differences in read depth or MAF among variants. These results are now shown in Figure 2D–E.

**Comment 3:** The p-values for XGBoost vs GLM were derived from a paired t-test, but CV folds share training data, violating independence. Bootstrap might be more accurate.

We agree with the reviewer and have replaced the cross-validation resample paired t-test with a paired comparison performed on the test and validation sets, which were excluded from training. For each model pair at a given threshold, we compute the ROC AUC from the held-out predictions and test the AUC difference using a paired bootstrap test (10,000 stratified resamples) and the DeLong test, both of which account for the same SNPs being scored by both models and therefore avoid the shared-training-data dependence of the fold-wise t-test. At the p < 1e-5 threshold, XGBoost significantly outperformed the linear models on both the test and validation sets [XGBoost vs GLM: ΔAUC = 0.0346, 95% bootstrap CI 0.0154-0.0545, bootstrap p = 5.45e-4, DeLong p = 4.83e-4; XGBoost vs GLMNET: ΔAUC = 0.0569, 95% CI 0.0370-0.0777, bootstrap p = 2.44e-8, DeLong p = 2.55e-8]. The revised Methods (Model evaluation and comparison) describe this procedure. We have replaced the cross-validation resample paired t-test with a paired bootstrap and DeLong comparison of the AUC difference on the test and validation sets (held-out SNPs) (Methods, Model evaluation and comparison).

**Comment 4:** All conclusions rest on a single small cohort and a single model; “selection” claims require independent supporting evidence. At minimum, an orthogonal computational validation (no new experiments) would be appreciated.

We thank the reviewer for this insightful comment. We have softened the selection language to “consistent with selective constraint” and have foregrounded the cohort and single-model limitations. We agree that an orthogonal computational validation would strengthen the work. As an orthogonal computational analysis, we tested whether the relationship between HNF4A-related model predictions and predicted deleteriousness differed between cluster 3 and the other clusters using CADD PHRED scores. We fit a linear regression model:

CADD phred score ~ cluster 3 * HNF4A + HNF1A + NEUROD1 + NKX6-1 + PDX1

Coefficients are shown in the table below:

We find that, while cluster 3 alone is not associated with differences in CADD PHRED scores, HNF4A SHAP scores and the interaction between HNF4A and cluster 3 are significantly associated with higher CADD PHRED scores (estimate = 1.56 ± 0.31, p = 6.05e-7; and estimate = 6.30 ± 2.43, p = 9.49e-3, respectively). Thus, increases in HNF4A binding-disruption SHAP scores were associated with larger increases in CADD PHRED scores for variants in cluster 3 compared with variants in the other clusters; the estimated relationship was approximately fivefold steeper in cluster 3. We added this analysis to the Results and cite it as orthogonal computational support for the cautious statement that the findings are “consistent with selective constraint,” rather than as causal evidence.

## Reviewer #3 — Minor points

**Comment 5:** Page 3, second-to-last line: the citation looks wrong — should be [6–9]?

Corrected: “[6,7,7–9]” has been fixed to “[6–9].”

**Comment 6:** Page 4: “transcription factors (TFs)” is already defined on page 3.

Corrected: the redundant re-definition has been removed (now “where TFs are bound”).

**Comment 7:** Page 4: “single nucleotide polymorphisms (SNPs)” — SNP was used earlier, so it should not be defined here.

Corrected: the redundant re-definition has been removed (now “noncoding SNPs”); SNP is defined at first use in the Abstract.

**Comment 8:** Page 5: the references are not sequential (they go to [40,42] and [41,44] here).

Corrected. We verified and corrected references throughout the text and renumbered citations into order of first appearance.

**Comment 9:** Page 12, point #3: regularization addresses overfitting, not imbalance. This sentence should be removed or revised.

Corrected. We revised the sentence to state that XGBoost’s internal regularization mitigates overfitting rather than class imbalance, and that imbalance was addressed through imbalance-appropriate metrics (ROC AUC and AUPRC) and comparisons across model classes rather than by resampling. We have revised the class-imbalance sentence in Methods (Running predictions).

**Comment 10:** Page 14: inconsistent p-value formatting throughout (e.g., ×10 vs 1e-5).

We have standardized p-value notation throughout to a single convention: 1e-5.

**Comment 11:** Page 18: “Figure 2A-D” looks like it should be Figure 3.

Corrected: see Reviewer 1 minor points; all such references in that section now point to Figure 3.

**Comment 12:** Suggest combining all supplementary tables into a single file.

We have combined Supplementary Tables S1–S5 into a single supplementary workbook (one sheet per table) for the resubmission. We have kept Table S6 as a text file because of its size (37 Mb).

**Comment 13:** Data availability: please ensure all data underlying figures/results are provided, with numerical source data in spreadsheet form.

Raw snATAC-seq data are available via dbGaP (phs000924), as stated in the Availability of data and materials section. Per-variant normalized values, SHAP scores, and cluster assignments underlying Figure 3 are provided in Table S6, and model performance metrics underlying Figure 2 are in Table S3; we will ensure the numerical source data for all figure panels are provided as supporting spreadsheets in line with the PLOS Genetics data availability policy.
