# Supplementary material for: XGBoost-SHAP analysis prioritizes lower-frequency variants predicted to affect HNF4A binding motifs in pancreatic progenitors
Source: PLoS Genet. 2026 Nov 4;22(11):e1012361. doi: 10.1371/journal.pgen.1012361 (PMC13754212; doi:10.1371/journal.pgen.1012361)
Supplement: Peer review history

# Peer review: Decision Letter 0

**DOI:** 10.1371/journal.pgen.1012361.r001

**Authors:** John M. Greally, Quan Sun

11 Jul 2026

PGENETICS-D-26-00534

XGBoost-based machine learning model reveals that active HNF4A binding sites are enriched for low-frequency SNPs in pancreatic progenitor cells

PLOS Genetics

Dear Dr. D'Antonio,

Thank you for submitting your manuscript to PLOS Genetics. After careful consideration, we feel that it has merit but does not fully meet PLOS Genetics's publication criteria as it currently stands. Therefore, we invite you to submit a revised version of the manuscript that addresses the points raised during the review process.

Please submit your revised manuscript by Sep 09 2026 11:59PM. If you will need more time than this to complete your revisions, please reply to this message or contact the journal office at plosgenetics@plos.org. When you're ready to submit your revision, log on to https://www.editorialmanager.com/pgenetics/ and select the 'Submissions Needing Revision' folder to locate your manuscript file.

Please include the following items when submitting your revised manuscript:

* A letter that responds to each point raised by the editor and reviewer(s). You should upload this letter as a separate file labeled 'Response to Reviewers'. This file does not need to include responses to any formatting updates and technical items listed in the 'Journal Requirements' section below.

* A marked-up copy of your manuscript that highlights changes made to the original version. You should upload this as a separate file labeled 'Revised Manuscript with Track Changes'.

* An unmarked version of your revised paper without tracked changes. You should upload this as a separate file labeled 'Manuscript'.

If you would like to make changes to your financial disclosure, competing interests statement, or data availability statement, please make these updates within the submission form at the time of resubmission. Guidelines for resubmitting your figure files are available below the reviewer comments at the end of this letter.

As the corresponding author, your ORCID iD is verified in the submission system and will appear in the published article. PLOS supports the use of ORCID, and we encourage all coauthors to register for an ORCID iD and use it as well. Please encourage your coauthors to verify their ORCID iD within the submission system before final acceptance, as unverified ORCID iDs will not appear in the published article. Only the individual author can complete the verification step; PLOS staff cannot verify ORCID iDs on behalf of authors.

We look forward to receiving your revised manuscript.

Kind regards,

Quan Sun, Ph.D.

Guest Editor

PLOS Genetics

John Greally

Section Editor

PLOS Genetics

Aimée Dudley

Editor-in-Chief

PLOS Genetics

Anne Goriely

Editor-in-Chief

PLOS Genetics

**Additional Editor Comments (if provided):**

All the referees have raised concerns about the potential confounding between read depth and the ASE label, which may indicate the model performance reflects statistical detection power rather than genuine regulatory biology. The authors should perform additional analysis to convince the reviewers and the editors. In addition, some causal languages throughout the manuscript regarding active HNF4A binding and selective constraint may be too strong, as SHAP interactions reflect model explanations rather than direct evidence of TF binding or function. I would recommend the authors softening the tone.

**Journal Requirements:**

If the reviewer comments include a recommendation to cite specific previously published works, please review and evaluate these publications to determine whether they are relevant and should be cited. There is no requirement to cite these works unless the editor has indicated otherwise.

1) Please ensure that the CRediT author contributions listed for every co-author are completed accurately and in full.

At this stage, the following Authors/Authors require contributions: Matteo D'Antonio. Please ensure that the full contributions of each author are acknowledged in the "Add/Edit/Remove Authors" section of our submission form.

The list of CRediT author contributions may be found here: https://journals.plos.org/plosgenetics/s/authorship#loc-author-contributions

2) Please provide an Author Summary. This should appear in your manuscript between the Abstract (if applicable) and the Introduction, and should be 150-200 words long. The aim should be to make your findings accessible to a wide audience that includes both scientists and non-scientists. Sample summaries can be found on our website under Submission Guidelines:

https://journals.plos.org/plosgenetics/s/submission-guidelines#loc-parts-of-a-submission

3) We do not publish any copyright or trademark symbols that usually accompany proprietary names, eg ©,  ®, or TM  (e.g. next to drug or reagent names). Therefore please remove all instances of trademark/copyright symbols throughout the text, including:

- TM on pages: 6, and 39.

4) Your current Financial Disclosure states, "This work was supported by the National Library Training Grant T15LM011271, the National Institute of Diabetes and Digestive and Kidney Disease (NIDDK) F31DK131867, U01DK105541, DP3DK112155 and P30DK063491, the National Heart, Lung and Blood Institute (NHLBI) F31HL158198 and U01HL107442, and the National Human Genome Research Institute (NHGRI) R41HG008118. Additional support was also received from the California Institute for Regenerative Medicine (CIRM) grant GC1R-06673-B. This publication includes data generated at the UC San Diego IGM Genomics Center utilizing an Illumina NovaSeq 6000 that was purchased with funding from a National Institutes of Health SIG grant S10OD026929.". However, your funding information on the submission form not indicates S10OD026929.

Please indicate by return email the full and correct funding information for your study and confirm the order in which funding contributions should appear. Please be sure to indicate whether the funders played any role in the study design, data collection and analysis, decision to publish, or preparation of the manuscript.

5) We have amended your Competing Interest statement to comply with journal style. We kindly ask that you double check the statement and let us know if anything is incorrect.

**Reviewers' comments:**

Reviewer's Responses to Questions

**Comments to the Authors:**

**Please note here if the review is uploaded as an attachment.**

Reviewer #1: In this manuscript, the authors present an XGBoost based interpretable machine learning framework to predict allele-specific effects with snATAC-seq data. They generated training data from 7 iPSC-derived pancreatic progenitor cells, identified allele-specific effects on chromatin accessibility using binomial test, and compared XGBoost with (regularized) logistic regression in predicting effects. They identified that XGBoost showed the best prediction accuracy, especially on stringent ASE binary labels, with read depths, peak scores and SNP MAFs as strong predictors; UMAP clustering analysis on SNP SHAP values revealed group of low-MAF variants showing uniquely strong interaction with HNF4A-related features.

Overall the manuscript is clearly written and investigates on an interesting problem: prioritizing non-coding variants with limited snATAC-seq data and comparing the accuracy using linear or non-linear methods. Meanwhile, some concerns need to be addressed:

1. The strongest predictor in the full XGBoost model is read depth, followed by MAF and peak score. The authors argue that read depth is both a biological signal and a statistical power variable, but the current analysis does not sufficiently separate these two roles. Since the outcome is defined by a binomial p-value for allelic imbalance, variants with higher read depth are mechanically more likely to achieve stringent significance even for the same allelic effect size. This makes the improved performance at p < 1e-5 difficult to interpret as evidence that XGBoost is learning regulatory biology rather than the read depth effect. As a sensitivity analysis, the authors could use beta-binomial or allelic ratio models with read depth modeled explicitly, downsample high-depth variants to a common depth, or repeat the analysis within read-depth/peak-score-matched strata. It would also be important to show whether the HNF4A cluster persists when read depth and peak score are removed, residualized, or matched across clusters.

2. Regarding to the authors’ conclusion that cluster 3 had lowest MAF: since MAF is included as a model input and SHAP values for all features are used to construct the UMAP and clusters, the subsequent observation that one SHAP-defined cluster is enriched for low-MAF variants is not an independent discovery. The authors should clarify whether the low-MAF cluster persists when MAF and MAF-SHAP features are excluded from the UMAP + clustering.

3. The SHAP interaction results suggest that cluster 3 has strong interactions involving MAF and HNF4A PWM scores, and the authors interpret this as enrichment for variants impacting HNF4A binding. However, SHAP interactions are explanations of a trained predictive model, not direct evidence of TF binding or functional impact. The manuscript should avoid presenting this as established HNF4A binding biology unless supported by independent evidence. To strengthen their statement, hte authors should add direct analyses of HNF4A motif overlap, motif disruption, footprinting, or external HNF4A ChIP-seq/CUT&RUN/CUT&Tag data in a relevant pancreatic lineage if available. If such data are not available, the conclusion should be softened to “variants predicted to affect HNF4A motifs” rather than “active HNF4A binding sites.”

4. Beyond this main result, several statements imply that the model reveals functional variant effects, selective constraints, or mechanisms of MODY1 phenotypic variability. However, the model predicts p-value-defined allele-specific chromatin accessibility using features that include detectability-related variables. SHAP values and UMAP clusters can prioritize hypotheses, but they do not establish causality. While the limitations section acknowledges this, but the title, abstract, conclusion, and discussion still overstate the biological certainty. II would suggest revising the title and abstract to use more cautious wording, such as “XGBoost-SHAP analysis prioritizes low-MAF variants predicted to affect HNF4A motifs in pancreatic progenitor chromatin.” The current title’s phrase “active HNF4A binding sites are enriched” implies direct binding evidence that is not clearly demonstrated.

Minor points:

Page 5: cell type-specific analysis was not well-established: despite usage of snATAC-seq + cell type clustering, the main analysis was still cell-type-agnostic. Consider modifying relevant statements.

Figure 1: caption defined ASE as allele-specific expression, but in main text it was defined as allele-specific effect (and is chromatin accessibility, not expression); both ASE and predictors pointed to the XGBoost model, creating ambiguity. Probably mark ASE separately as the outcome.

Several figures have been referred correctly, such as in section UMAP clustering of SHAP values uncovers nonlinear interactions between SNP MAFs and HNF4A PWM scores, Figure 2 was referred (should be Figure 3)

Reviewer #2: Developing methods to prioritize functional variants is a critical need in interpreting GWAS associations. The current manuscript uses new approaches in machine learning to build predictive models that aim to move beyond black box prediction toward interpretable features. Towards that goal, the clustering and dimensional reduction to explore SHAP profiles is interesting and valuable. Further, the approach outlined uses allele-specific chromatin accessibility as a proxy for regulatory activity, and predicts it from variant features. By using single-cell ATAC-seq data specifically, this is an interesting and valuable approach, as it may allow nomination of variant function in a cell-type-specific manner not reachable by bulk sequencing methods. Further, the authors provide clear reporting of important variables in their models, what they tested, and how they interpreted the outcomes, which was genuinely helpful to read and interpret.

In the introduction the authors state “High read depth and peak scores are linked to SNPs in highly active functional elements, which are more likely to show ASE”. This is a bit circular, as I believe the assumption here is that they are highly active because they have high read depth. It seems that this claim would be more stable simplified to “SNPs in putative functional elements with high read depth and peak scores are more likely to show ASE”. I think this is a claim about clusters 5 and 7 in the UMAP, so perhaps a slightly different wording can make the claim clear; however, this is also related to the main concern for the manuscript to address.

It isn’t clear how sensitive the model is to sequencing depth or total number of cells needed in single-cell data. This is important for two reasons. First is the extendibility of this approach to data from fewer cells. If the hope is that this approach can be applied to single-cell data, yet the test case used in the manuscript represented >90% of the cells likely allowing increased read coverage per peak. The authors highlight the value of cell-type specificity (page 20). Second, this speaks to what was identified to be the most important feature. The authors clearly articulate that read depth has the largest SHAP values and is the most important variable in their model to accurately predict ASE. But read depth is inherently linked to the binomial test’s power. Likely because higher sequencing coverage allows detection of more reads at a given SNPs. In other words, deeply sequenced SNPs are more likely to have enough power to be called significant by the binomial test. And perhaps the model learns that the label is tied to read depth, which seems to be a property of statistical labeling rather than biology. The authors address this in several places, including on page 17 and when talking about Cluster 7 (page 18 second paragraph), but they dismiss it with an assertion (that promoter ASE reflects genuine activity) they do not back up with data. Read depth and function are expected be tied in chromatin accessibility data, it carries real biology and there is no argument there. It is also true that in their model the sequencing data beats the TF features using the position weight matrix, but that does not show that what the model predicts isn’t a confound with power.

It would strength the paper to provide an analysis that can distinguish the two interpretations. The one cluster that is defined as the highest read depth, is the one most exposed to the bias in detection. If promoters and non-promoters have equal effect sizes but promoters have higher depth, promoters will get labeled ASE-positive more often, because higher depth give the binomial test more power to call the same sized effect significant. But if at matched depth promoters have larger effect sizes than non-promoters, then promoters would be enriched among the positives even after depth advantage is removed.

Currently the conclusions seem to focus on the association with HNF4A, but the paper is largely about building a model to predict variants that are functional based on impact on chromatin accessibility. Do the authors want to make any conclusions about the method to develop the model?

The manuscript mentions the imbalance in the data for ASE positive SNPs, and rightly reports AUPRC. But the AUPRC is only reported in the supplemental table 3. The class imbalance makes the ROC AUC stay high despite the large number of false positives required to capture the true positives, because those false positives have little effect on the false-positive rate (large denominator). The manuscript could be further strengthened by reporting AUPRC as fold-over-baseline when performance is claimed so that the magnitude of the improvement is clear, which is the case in their data for the XGB model.

In the discussion about HNF4A, the authors state that the low frequency variants in cluster 3 have modest impact. Do the authors mean modest in a low population burden or modest impact on ASE? The following sentence says that variants in the binding sites have pronounced effect on transcriptional regulation. It would help if the authors could clarify these differences.

With Figure 2D. While the text is clear what went into the sequence or TF only, it might help to mention in the figure legend that sequence only is the sequence related data only. It can be misinterpreted to mean sequence of the DNA instead of read depth, peak score, and MAF.

Reviewer #3: The authors generate single-nucleus ATAC-seq data from seven iPSC-derived pancreatic progenitor cell samples. They train XGBoost, logistic regression, and regularized logistic regression models to predict allele-specific effects from chromatin read depth, peak score, MAF, and PWM predictions for five pancreatic TFs, reporting that XGBoost outperforms the linear models at stringent thresholds. Using SHAP-based interpretation and UMAP, they identify a cluster of lower-MAF variants with strong predicted interactions at HNF4A binding sites, which they interpret as evidence of selective constraint on HNF4A binding in pancreatic progenitors. This paper is well-written while I have some concerns:

Major:

- I'm a little confused about the "low frequency" claims, since the authors already filtered MAF > 5% which is the definition of common variants in my concept

- While the authors claim that "read depth in accessible chromatin regions is itself a biological signal", I would recommend the authors to do a sensitivity check by controlling the read depth. For example bin SNPs into read-depth deciles and recompute ROC AUC and AUPRC within each stratum. If within-stratum AUC remains meaningfully above 0.5 and XGBoost still over the linear models, the biological signal is supported. Otherwise if it collapses toward 0.5, the apparent performance is attributable to depth differences between SNPs and you should interpret the results with cautious.

- The pvalues for XGBoost and glm comparsion were dervied from paired ttest. But the cv folds share training data, violating the independence assumption of the paired t-test. Boostrap might be a more accurate choice.

- All conclusions are associated to a single small cohort and a single model, while the "selection" claims require independent supporting evidence. It would be appreciated if the authors could provide, at minimum, an orthogonal computational validation, new experiments are not needed.

Minor:

Page 3: second to the last row, citation looks weird? should be [6-9]?

Page 4: "transcription factors (TFs)" already defined in page3

Page 4: "single nucleotide polymorphisms (SNPs)" you already used "SNP" in previous text, you shouldn't define here

Page 5: again, your reference looks not sequential, since the reference go up to "[40,42]" and "[41,44]" here. You should double check your reference.

Page 12: point #3, regularization addresses overfitting, not imbalance. This sentence should be removed or revised.

Page 14: you had inconsistent pvalues format through the entire manuscript (i.e., x10 and 1e-5)

Page 18: "Figure 2A-D" -> looks like you are referring to Figure 3?

I would suggest putting all supp tables into one single file for convenience.

**********

**Have all data underlying the figures and results presented in the manuscript been provided?**

Large-scale datasets should be made available via a public repository as described in the *PLOS Genetics*  data availability policy, and numerical data that underlies graphs or summary statistics should be provided in spreadsheet form as supporting information.

Reviewer #1: Yes

Reviewer #2: Yes

Reviewer #3: Yes

**********

PLOS authors have the option to publish the peer review history of their article (what does this mean?). If published, this will include your full peer review and any attached files.

If you choose “no”, your identity will remain anonymous but your review may still be made public.

**Do you want your identity to be public for this peer review?**  For information about this choice, including consent withdrawal, please see our Privacy Policy.

Reviewer #1: No

Reviewer #2: No

Reviewer #3: No

[NOTE: If reviewer comments were submitted as an attachment file, they will be attached to this email and accessible via the submission site. Please log into your account, locate the manuscript record, and check for the action link "View Attachments". If this link does not appear, there are no attachment files.]

**Figure resubmission:**

While revising your submission, we strongly recommend that you use PLOS’s NAAS tool (https://ngplosjournals.pagemajik.ai/artanalysis) to test your figure files. NAAS can convert your figure files to the TIFF file type and meet basic requirements (such as print size, resolution), or provide you with a report on issues that do not meet our requirements and that NAAS cannot fix.

After uploading your figures to PLOS’s NAAS tool - https://ngplosjournals.pagemajik.ai/artanalysis, NAAS will process the files provided and display the results in the "Uploaded Files" section of the page as the processing is complete. If the uploaded figures meet our requirements (or NAAS is able to fix the files to meet our requirements), the figure will be marked as "fixed" above. If NAAS is unable to fix the files, a red "failed" label will appear above. When NAAS has confirmed that the figure files meet our requirements, please download the file via the download option, and include these NAAS processed figure files when submitting your revised manuscript.

**Reproducibility:**

To enhance the reproducibility of your results, we recommend that authors of applicable studies deposit laboratory protocols in protocols.io, where a protocol can be assigned its own identifier (DOI) such that it can be cited independently in the future. Additionally, PLOS ONE offers an option to publish peer-reviewed clinical study protocols. Read more information on sharing protocols at https://plos.org/protocols?utm_medium=editorial-email&utm_source=authorletters&utm_campaign=protocols

# Author comment: Author response to Decision Letter 1

**DOI:** 10.1371/journal.pgen.1012361.r002

4 Sep 2026

Attachment Submitted filename: Response_to_Reviewers.V12.docx

# Peer review: Decision Letter 1

**DOI:** 10.1371/journal.pgen.1012361.r003

**Authors:** John M. Greally, Quan Sun

30 Sep 2026

PGENETICS-D-26-00534R1

XGBoost-SHAP analysis prioritizes lower-frequency variants predicted to affect HNF4A binding motifs in pancreatic progenitors

PLOS Genetics

Dear Dr. D'Antonio,

Thank you for submitting your manuscript to PLOS Genetics. After careful consideration, we feel that it has merit but does not fully meet PLOS Genetics's publication criteria as it currently stands. Therefore, we invite you to submit a revised version of the manuscript that addresses the points raised during the review process.

Please submit your revised manuscript within by Oct 30 2026 11:59PM. If you will need more time than this to complete your revisions, please reply to this message or contact the journal office at plosgenetics@plos.org. When you're ready to submit your revision, log on to https://www.editorialmanager.com/pgenetics/ and select the 'Submissions Needing Revision' folder to locate your manuscript file.

Please include the following items when submitting your revised manuscript:

* A letter that responds to each point raised by the editor and reviewer(s). You should upload this letter as a separate file labeled 'Response to Reviewers'. This file does not need to include responses to formatting updates and technical items listed in the 'Journal Requirements' section below.

* A marked-up copy of your manuscript that highlights changes made to the original version. You should upload this as a separate file labeled 'Revised Manuscript with Track Changes'.

* An unmarked version of your revised paper without tracked changes. You should upload this as a separate file labeled 'Manuscript'.

If you would like to make changes to your financial disclosure, competing interests statement, or data availability statement, please make these updates within the submission form at the time of resubmission. Guidelines for resubmitting your figure files are available below the reviewer comments at the end of this letter.

As the corresponding author, your ORCID iD is verified in the submission system and will appear in the published article. PLOS supports the use of ORCID, and we encourage all coauthors to register for an ORCID iD and use it as well. Please encourage your coauthors to verify their ORCID iD within the submission system before final acceptance, as unverified ORCID iDs will not appear in the published article. Only the individual author can complete the verification step; PLOS staff cannot verify ORCID iDs on behalf of authors.

We look forward to receiving your revised manuscript.

Kind regards,

Quan Sun, Ph.D.

Guest Editor

PLOS Genetics

John Greally

Section Editor

PLOS Genetics

Aimée Dudley

Editor-in-Chief

PLOS Genetics

Anne Goriely

Editor-in-Chief

PLOS Genetics

**Additional Editor Comments (if provided):**

Reviewer #1 still has a minor concern of the languages and suggests to temper the statement. We will be happy to accept the manuscript after this small fix.

**Journal Requirements:**

If the reviewer comments include a recommendation to cite specific previously published works, please review and evaluate these publications to determine whether they are relevant and should be cited. There is no requirement to cite these works unless the editor has indicated otherwise.

1) We have noticed that you have uploaded Supporting Information files, but you have not included a list of legends. Please add a full list of legends for your Supporting Information files after the references list.

**Reviewers' comments:**

Reviewer's Responses to Questions

**Comments to the Authors:**

**Please note here if the review is uploaded as an attachment.**

Reviewer #1: The authors have substantially addressed my concern regarding the potential confounding effect of read depth. I appreciate the addition of the read-depth-stratified analyses, in which XGBoost, GLM, and GLMNET were independently trained and evaluated within read-depth deciles, as well as the corresponding MAF-stratified analysis. One point could still be clarified further. The additional regression analysis shows that the association between MAF and cluster 3 membership remains after adjustment for read depth and peak score, which is informative. However, this does not strictly demonstrate that the cluster itself, or its HNF4A-related interaction pattern, is independent of read depth and peak score. I therefore suggest slightly tempering statements such as “cluster 3’s MAF properties were independent of read depth and peak score” and instead stating more precisely that the association between MAF and cluster 3 membership remained after adjustment for these variables.

Reviewer #2: The authors have done a commendable job addressing my comments. They added analyses of model performance across read-depth strata, which were requested independently by the other reviewers and provide greater context for the performance of their approach. They have also tempered the interpretation of the cluster 7 result to note that detection power and biological effect are not separable with the current data, which addresses my concern. The conclusions and phrasing now better reflect the support in the data. I have no further concerns and recommend acceptance.

Reviewer #3: The authors have addressed my comments

**********

**Have all data underlying the figures and results presented in the manuscript been provided?**

Large-scale datasets should be made available via a public repository as described in the *PLOS Genetics*  data availability policy, and numerical data that underlies graphs or summary statistics should be provided in spreadsheet form as supporting information.

Reviewer #1: Yes

Reviewer #2: Yes

Reviewer #3: None

**********

PLOS authors have the option to publish the peer review history of their article (what does this mean?). If published, this will include your full peer review and any attached files.

If you choose “no”, your identity will remain anonymous but your review may still be made public.

**Do you want your identity to be public for this peer review?**  For information about this choice, including consent withdrawal, please see our Privacy Policy.

Reviewer #1: No

Reviewer #2: No

Reviewer #3: No

[NOTE: If reviewer comments were submitted as an attachment file, they will be attached to this email and accessible via the submission site. Please log into your account, locate the manuscript record, and check for the action link "View Attachments". If this link does not appear, there are no attachment files.]

**Figure resubmission:**

While revising your submission, we strongly recommend that you use PLOS’s NAAS tool (https://ngplosjournals.pagemajik.ai/artanalysis) to test your figure files. NAAS can convert your figure files to the TIFF file type and meet basic requirements (such as print size, resolution), or provide you with a report on issues that do not meet our requirements and that NAAS cannot fix.

After uploading your figures to PLOS’s NAAS tool - https://ngplosjournals.pagemajik.ai/artanalysis, NAAS will process the files provided and display the results in the "Uploaded Files" section of the page as the processing is complete. If the uploaded figures meet our requirements (or NAAS is able to fix the files to meet our requirements), the figure will be marked as "fixed" above. If NAAS is unable to fix the files, a red "failed" label will appear above. When NAAS has confirmed that the figure files meet our requirements, please download the file via the download option, and include these NAAS processed figure files when submitting your revised manuscript.

**Reproducibility:**

To enhance the reproducibility of your results, we recommend that authors deposit laboratory protocols in protocols.io, where a protocol can be assigned its own identifier (DOI) such that it can be cited independently in the future. Additionally, PLOS ONE offers an option to publish peer-reviewed clinical study protocols. Read more information on sharing protocols at https://plos.org/protocols?utm_medium=editorial-email&utm_source=authorletters&utm_campaign=protocols

# Author comment: Author response to Decision Letter 2

**DOI:** 10.1371/journal.pgen.1012361.r004

20 Oct 2026

Attachment Submitted filename: response2_V02.docx

# Editor report: Decision Letter 2

**DOI:** 10.1371/journal.pgen.1012361.r005

**Authors:** John M. Greally, Quan Sun

21 Oct 2026

Dear Dr D'Antonio,

We are pleased to inform you that your manuscript entitled "XGBoost-SHAP analysis prioritizes lower-frequency variants predicted to affect HNF4A binding motifs in pancreatic progenitors" has been editorially accepted for publication in PLOS Genetics. Congratulations!

Before your submission can be formally accepted and sent to production you will need to complete our formatting changes, which you will receive in a follow up email. Please be aware that it may take several days for you to receive this email; during this time no action is required by you. Please note: the accept date on your published article will reflect the date of this provisional acceptance, but your manuscript will not be scheduled for publication until the required changes have been made.

Once your paper is formally accepted, an uncorrected proof of your manuscript will be published online ahead of the final version, unless you’ve already opted out via the online submission form. If, for any reason, you do not want an earlier version of your manuscript published online or are unsure if you have already indicated as such, please let the journal staff know immediately at plosgenetics@plos.org.

In the meantime, please log into Editorial Manager at https://www.editorialmanager.com/pgenetics/, click the "Update My Information" link at the top of the page, and update your user information to ensure an efficient production and billing process. Note that PLOS requires an ORCID iD for all corresponding authors. Therefore, please ensure that you have an ORCID iD and that it is validated in Editorial Manager. To do this, go to ‘Update my Information’ (in the upper left-hand corner of the main menu), and click on the Fetch/Validate link next to the ORCID field.  This will take you to the ORCID site and allow you to create a new iD or authenticate a pre-existing iD in Editorial Manager.

If you have a press-related query, or would like to know about making your underlying data available (as you will be aware, this is required for publication), please see the end of this email. If your institution or institutions have a press office, please notify them about your upcoming article at this point, to enable them to help maximise its impact. Inform journal staff as soon as possible if you are preparing a press release for your article and need a publication date.

Thank you again for supporting open-access publishing; we are looking forward to publishing your work in PLOS Genetics!

Yours sincerely,

Quan Sun, Ph.D.

Guest Editor

PLOS Genetics

John Greally

Section Editor

PLOS Genetics

Aimée Dudley

Editor-in-Chief

PLOS Genetics

Anne Goriely

Editor-in-Chief

PLOS Genetics

www.plosgenetics.org

BlueSky: @plos.bsky.social

----------------------------------------------------

Comments from the reviewers (if applicable):

----------------------------------------------------

**Data Deposition**

If you have submitted a Research Article or Front Matter that has associated data that are not suitable for deposition in a subject-specific public repository (such as GenBank or ArrayExpress), one way to make that data available is to deposit it in the Dryad Digital Repository. As you may recall, we ask all authors to agree to make data available; this is one way to achieve that. A full list of recommended repositories can be found on our website.

The following link will take you to the Dryad record for your article, so you won't have to re‐enter its bibliographic information, and can upload your files directly:

http://datadryad.org/submit?journalID=pgenetics&manu=PGENETICS-D-26-00534R2

More information about depositing data in Dryad is available at http://www.datadryad.org/depositing. If you experience any difficulties in submitting your data, please contact help@datadryad.org for support.

Additionally, please be aware that our data availability policy requires that all numerical data underlying display items are included with the submission, and you will need to provide this before we can formally accept your manuscript, if not already present.

----------------------------------------------------

**Press Queries**

If you or your institution will be preparing press materials for this manuscript, or if you need to know your paper's publication date for media purposes, please inform the journal staff as soon as possible so that your submission can be scheduled accordingly. Your manuscript will remain under a strict press embargo until the publication date and time. This means an early version of your manuscript will not be published ahead of your final version. PLOS Genetics may also choose to issue a press release for your article. If there's anything the journal should know or you'd like more information, please get in touch via plosgenetics@plos.org.

# Editor report: Acceptance letter

**DOI:** 10.1371/journal.pgen.1012361.r006

**Authors:** John M. Greally, Quan Sun

PGENETICS-D-26-00534R2

XGBoost-SHAP analysis prioritizes lower-frequency variants predicted to affect HNF4A binding motifs in pancreatic progenitors

Dear Dr D'Antonio,

We are pleased to inform you that your manuscript entitled "XGBoost-SHAP analysis prioritizes lower-frequency variants predicted to affect HNF4A binding motifs in pancreatic progenitors" has been formally accepted for publication in PLOS Genetics! Your manuscript is now with our production department and you will be notified of the publication date in due course.

The corresponding author will soon be receiving a typeset proof for review, to ensure errors have not been introduced during production. Please review the PDF proof of your manuscript carefully, as this is the last chance to correct any errors. Please note that major changes, or those which affect the scientific understanding of the work, will likely cause delays to the publication date of your manuscript.

Soon after your final files are uploaded, unless you have opted out or your manuscript is a front-matter piece, the early version of your manuscript will be published online. The date of the early version will be your article's publication date. The final article will be published to the same URL, and all versions of the paper will be accessible to readers.

For Research Articles, you will receive an invoice from PLOS for your publication fee after your manuscript has reached the completed accept phase. If you receive an email requesting payment before acceptance or for any other service, this may be a phishing scheme. Learn how to identify phishing emails and protect your accounts at https://explore.plos.org/phishing.

Thank you again for supporting PLOS Genetics and open-access publishing. We are looking forward to publishing your work!

With kind regards,

Sharmila Kamatchi

PLOS Genetics

On behalf of:

The PLOS Genetics Team

Carlyle House, Carlyle Road, Cambridge CB4 3DN | United Kingdom

plosgenetics@plos.org | +44 (0) 1223-442823

plosgenetics.org | Twitter: @PLOSGenetics
